# Supplementary material for: Reconciling crop production, climate action and nature conservation in Europe by agricultural intensification and extensification
Source: Nat Commun. 2025 Nov 21;16:10289. doi: 10.1038/s41467-025-65201-4 (PMC12638777; doi:10.1038/s41467-025-65201-4)
Supplement: Supplementary file 1 — Supplementary Information [file 41467_2025_65201_MOESM1_ESM.pdf]

Supplementary Text 1 | Identification of low productive cropland

Low productive cropland is identified using cumulative normalized difference vegetation index (NDVI) over the growing season as a proxy for agricultural productivity across 164 subregions (see **Supplementary Fig. 9** for a representation of the subregions and **Supplementary Fig. 21** for a schematic description of the approach). NDVI is a common indicator for crop biomass monitoring and frequently used to estimate crop yields[1-3]. NDVI data were obtained from the Terra Moderate Resolution Imaging Spectroradiometer Vegetation Indices dataset (MOD13Q1.061) with 250 m resolution[4]. The Savitzkey-Golay (SG) filter is applied to smooth the NDVI series, with the smoothing windows' half-width of 4. Pixels with a quality reliability (from "SummaryQA" band of MOD13Q1.061) of less than or equal to 1 are considered useful, while those with a higher value are replaced with the SG-filtered value. The growing season in each cropland pixel was determined by the crop phenology data from the Anomaly Hotspots of Agricultural Production (ASAP) dataset with 500 m resolution[5]. It includes start of the growing season (SOS), end of growing season (EOS), growing season length and number of growing seasons per year, based on the long-term average of 10-day MODIS/Visible Infrared Imager Radiometer Suite (VIIRS) Fraction of Photosynthetically Active Radiation absorbed by vegetation (FPAR) data. The date when FPAR reaches 25% of the ascending amplitude of the season, and when it drops below 35%, are defined as the SOS and the EOS, respectively. By integrating the crop phenology and the NDVI data, we calculated the NDVI sum of the corresponding growing season between 2018 and 2022. This approach can reduce the impact of short-term climate fluctuations, and consider the growth season difference of various crops, such as winter wheat and spring wheat. In order to account for the differences in the crop growing cycles and agricultural technology across Europe, we perform this analysis for specific subregions by integrating 31 ecoregions[6] with 41 administrative regions (**Supplementary Fig. 9**), where each subregion largely shares similar conditions of climate, soil, geology, and cultivation technology. The z-score method was used to determine the low productive threshold in each subregion. According to the ranking of cropland relative abundance in the 164 subregions, defined as the cropland area relative to the total land area in each subregion, the values of z-score ranging between -2 and -0.5 following a normal distribution centered at -1.25 were assigned to different subregions. For example, subregions in France with higher cropland relative abundancy are assigned a z-score closer to -2, while subregions in Norway, where cropland abundancy is lower, are assigned a z-score closer to -0.5. The z-score is then used to identify the threshold of low productivity indicator, i.e., the growing season total NDVI specific to each subregion. consequently, land abundance is associated with a higher z-score and

consequently a higher fraction of low productivity cropland within that subregion. In addition, the growing season average NDVI of all pure cropland pixels are assessed at a European level and the lower quartile (25 percentile) is used as another low productivity threshold. Only cropland pixels that meet both conditions above (i.e., cropland relative abundance and lower-than-25-percentile NDVI of the growing season at European level) are classified as low productivity pixels. The identification of low productivity cropland is restricted to pure cropland pixels with single growing season (i.e., with one crop produced per year), to avoid the noise in NDVI records introduced by the natural vegetation component in mosaic cropland and multiple growing seasons.

Supplementary Text 2 | Assessment of yield effects of mosaic cropland

Compared to pure cropland, the presence of non-crop vegetation (i.e., multifunctional landscapes) affects the crop yields in the mosaic cropland through microclimate regulation, pest control, and improved soil conditions[7,8]. In this analysis, we estimated the relative impact of non-crop vegetation within mosaic cropland on yields in each subregion by comparing the present-day yields between pure and mosaic cropland (the schematic of the calculation is shown in **Supplementary Fig. 22**). A higher resolution (4 km) wheat yield dataset[9] was used to unmix the multifunctional landscapes' impact on crop yield of mosaic cropland. This is the yield dataset with the highest resolution in Europe, and its validation has shown high overall consistency with observed yields ($R^2 = 0.78$). In idealized terms, the contributions to crop yield in each pixel can be decomposed into the one from the cropland itself and the indirect benefit from the vegetation on the non-cropland area of the mosaic pixels**:**

$$Y_{obs} = (1 - \partial) \times Y_P + \partial Y_M \qquad \textbf{(1)}$$

Where ∂ is the fraction of mosaic cropland to total cropland in each 4-km resolution pixel, and 1-∂ is the fraction of pure cropland to total cropland, which is based on the ESA-CCI land cover data. Y_{obs} is the 4-km resolution pixel's yield, Y_P is the yield in pure croplands, and Y_M is the yield in the mosaic croplands. Y_M is affected by the presence of non-crop vegetation in proximity to cropland, because trees, shrubs, or grass may produce either positive or negative effects on crop yields by interacting with soil fertility and competition for resources such as sunlight, water, and nutrients. The Y_{obs} values and the corresponding fraction of mosaic cropland relative to total cropland area (∂) were sourced from this yield data and ESA-CCI data (yellow points in **Supplementary Fig. 22b**).

A zero-impact straight line (the red line in **Supplementary Fig. 22b**) represents the situation in which the crop yields are unaffected by the vegetation in the non-cropland fraction of the mosaic pixel. It is defined by the yield of fully pure cropland ($\partial = 0$, Y_P), and the yield of fully mosaic cropland ($\partial = 1$, 50% of the Y_P, assumed that mosaic cropland is composed of 50% cropland and 50% non-crops vegetation).

$$Y_{zi} = (1 - 0.5 \times \partial) \times Y_P \qquad \textbf{(2)}$$

Where Y_{zi} is the yield along the zero-impact straight line under varying fractions of pure cropland, representing the case in which the non-crop vegetation of the mosaic pixel does not affect crop yields. The difference between Y_{obs} and Y_{zi} reflects the impact of non-crop vegetation on crop yields within mosaic cropland pixels. Any points above the zero-impact line indicate positive impacts of non-crop vegetation on yields, whereas any points below the zero-impact line indicate negative impacts. Hence, the relative impact

(ω) of non-crop vegetation within mosaic cropland on yields can be calculated as

$$\omega = \frac{Y_{obs} - Y_{zi}}{Y_{zi}} \quad \textbf{(3)}$$

The relative impact (ω) of non-crop vegetation within mosaic cropland on yields was quantified across 164 subregions. Y_P was determined by the mean yield of the upper tenth (i.e., top 10%) percentile of pure cropland samples and corresponding fraction of pure cropland. The remaining observation data of Y_{obs} were compared with the corresponding Y_{zi}, and then the mean difference was used to quantify the impact of non-crop vegetation on yield of mosaic cropland (i.e., ω) in each subregion. We do not quantify ω through a direct comparison of the 4-km-resolution yield difference between pixels of fully pure cropland and fully mosaic cropland because of the low number of available observations for these two pure land cover types in the resolution of the yield dataset. This modeling is performed only when there are more than 50 stable yield observations available in the subregion to secure a robust estimate. Spring wheat yields are mainly used to estimate ω. Only when the spring wheat samples are insufficient (< 50 samples), winter wheat yield data are alternatively used. Across all subregions, we used a z-score of '-2' and '2' as the thresholds to exclude extreme estimates and outliers in the yield dataset. For subregions lacking robust estimates and sufficient samples (accounting for 19.6% of European total area), the average value of ω from neighboring subregions is used as a substitute. Then, ω was used to estimate the crop yield of the cropland areas within the mosaic cropland pixels, including the new mosaic cropland resulting from extensification and the crop losses from the revegetation of suboptimal mosaic cropland, as well as the relative reduction in yields of the cropland share in mosaic cropland converted to pure cropland during intensification.

Supplementary Text 3 | Uncertainties and limitations

The spatial patterns of suboptimal cropland identified here broadly align with previous estimates of unsustainable cropland characterized by high soil erosion rates and steep slopes (**Supplementary Tab. 6**), whose total estimates range from about 20 to 50 million hectares (Mha) in Europe. Higher values are achieved when climate and social aspects are factored in, while the lower end mostly reflects biophysical considerations. These discrepancies between estimates are due to the use of different identification criteria, input datasets, and spatial-temporal mismatches[10,11]. A sensitivity test (**Supplementary Tab. 5**) using different identification thresholds of suboptimal cropland indicates that the estimated area can vary from 20.6 Mha (lower-bound, **Supplementary Fig. 17**) to 29.5 Mha (upper-bound, **Supplementary Fig. 18**), with spatial patterns that are broadly consistent with the estimate (24.2 Mha) presented in the main text (**Fig. 2**).

Our analysis found general yield benefits in presence of non-crop vegetation within mosaic cropland. A European average increase of 9.49% (-1.41% and 24.9% as the 5th and 95th percentile), relative to average yields of pure cropland, is estimated (**Supplementary Fig. 10**). This value is slightly lower than observations based on individual plot surveys (**Supplementary Tab. 1**). The reason can be that plot surveys typically investigate well-managed and diversified patches of agricultural land and tree species, whereas in most of the existing mosaic cropland the mix of crops and trees is not optimized and the yield-increase effect is more limited[12,13]. Some negative impacts are also observed in northern Europe, eastern Europe, and semi-arid regions of Spain, where crop growth can potentially be constrained by competition with trees for sunlight, nutrients and water resources, as reported elsewhere[12-14]. Implementation of extensification strategies in these locations needs careful consideration of the local context and selection of tree species that reduce risks of competition for resources with crops.

In the implementation of the crop switching strategies, it is assumed that the newly cultivated crops are fully harvested, in line with ref[15-17]. These are large-scale field crops suitable for advanced mechanized production and are located in areas with high levels of agricultural intensification and favorable cultivation conditions (i.e., high NPCD). As a sensitivity test, we also consider an 80% harvest rate for both intensification and extensification scenarios, representing suboptimal growing conditions or harvesting from 80% of the cultivation area (with the remaining 20% accounting for crop failure or land used for auxiliary infrastructure, **Supplementary Fig. 24**). Results show that, despite a larger area for cropland use mode conversion is needed, I-HC (6.89 Mha), E-HC (22.8 Mha), and E-HS (45.0 Mha) scenarios can still fully offset the calorie losses from revegetating suboptimal cropland, whereas the I-HS scenario does not achieve

full compensation (68.7%). This is consistent with the main conclusions of the study.

There are uncertainties related to the estimates of crop yields and carbon sequestration rates from natural vegetation regrowth and afforestation. Crop yields are estimated with GAEZ+2015, a database that combines national and sub-national crop and irrigation statistics with the support of detailed spatial multisource data, including cropland, irrigation, soil properties, climate, human activity, and observed crop phenology and crop calendars. The database has been validated against other yield models and observations[18,19], and it is among the most widely applied in agricultural studies[16,17,20]. The impact of crop-specific yield fluctuations on the area required for cropland intensification and extensification was investigated in an uncertainty analysis (**Supplementary Fig. 19**), which shows that the suboptimal cropland can provide a total calorie supply ranging from 55.8 Pcal to 69.2 Pcal (61.3 Pcal reported in the main results). The required total areas for cropland intensification (4.76-6.84 Mha for upper-bound estimates and 4.80-6.84 Mha for lower-bound estimates) or extensification (16.6-32.2 Mha (upper) and 17.1-31.5 Mha (lower)) change accordingly, but their spatial patterns remain largely similar to those described in the main text.

Uncertainty ranges in the estimates of climate change mitigation potentials from the investigated scenarios are presented in **Fig. 6**. These ranges include uncertainties in the dataset used to estimate each source of carbon flow (carbon sequestration from trees in the revegetated suboptimal cropland and in the extensification scenarios, emissions from clearing land and afforestation activities, and fertilization-induced N_2O emissions). Because the available datasets express their uncertainties in incompatible metrics, a uniform error-propagation scheme was not feasible. Instead, we constructed a conservative envelope to estimate the aggregated uncertainty of the datasets used through pairing the lowest-bound estimate (mean - uncertainty) of carbon sequestration, with the highest-bound estimate (mean + uncertainty) of carbon emissions, and vice versa.

More specifically, rates of natural vegetation regrowth are sourced from a study that produced a spatial--explicit dataset by gathering more than 13,000 georeferenced measurements of carbon accumulation, and it has been extensively validated in the original publication[21]. The ensemble model that created the dataset had an RMSE of 0.798 Mg C ha^{-1} yr^{-1} and an R^2 of 0.445 on an independent test set. In this analysis, the average sequestration potential of natural regrowth in suboptimal cropland is 4.10 ± 1.08 t CO_2 ha^{-1} yr^{-1}. This estimate is close to those from studies at similar latitude (full range from 2.57 to 5.50 t CO_2 ha^{-1} yr^{-1}, **Supplementary Tab. 7**), but it is lower than the global average (full range from 11.6 to 13.1 t CO_2 ha^{-1} yr^{-1}), as sequestration rates in the tropics can be an order of magnitude higher than those at mid or high latitudes[21].

Carbon sequestration rates for afforestation are based on the Global Forest Model (G4M)[22,23], a well-established forest model linked to Integrated Assessment Models for estimating land use change and forestry emissions for various SSP-RCP scenarios[24,25]. The model is based on satellite retrievals of net primary production and national statistics, and it is validated against observations and multi-model intercomparison studies[26,27]. Relative to other forest models, G4M shows better skills in reproducing mean annual increments of forests in Europe, for which biomass stock estimated from G4M are close to those from statistical observations (16,400 Tg vs. 16,800 Tg). At a country level, spatial data of G4M are broadly consistent with empirically-derived estimates, and show smaller relative errors than other forest models ($R^2 = 0.79$), with a small negative bias (i.e., underestimation of forest biomass density)[26]. In our analysis, the carbon sequestration potential of afforestation in suboptimal cropland is 9.83±2.36 t CO_2 ha^{-1}, and the sequestration potential of planting coniferous and non-coniferous forest is 11.0±1.67 t CO_2 ha^{-1} yr^{-1} and 8.90±2.45 t CO_2 ha^{-1} yr^{-1}, respectively. These estimates of carbon sequestration are close to those from other studies (full range from 6.45 to 11.1 t CO_2 ha^{-1} yr^{-1}, **Supplementary Tab. 8**). The average carbon sequestration of vegetation fraction in new mosaic cropland is 4.80±2.16 t CO_2 ha^{-1} yr^{-1}.

The land use transitions of our study do not include estimates of changes in soil organic carbon (SOC). Robust datasets of long-term SOC changes following natural vegetation regrowth, afforestation, or crop switching are currently unavailable. Both positive and negative changes in SOC are reported, depending on specific local factors, climatic conditions, and experiment (e.g., temporal coverage and soil depth). Timeseries of SOC changes are usually available for cropland remaining cropland or converted to perennial grassland[28,29], but uncertainties are high and attribution of SOC changes to individual shifts in cultivation from one crop type to another remains challenging. Average carbon changes in soils after natural vegetation regrowth are found to positively influence SOC but the benefits vary greatly and, in some cases, they can be negligible[21,30]. Adopting a low-confident average SOC sequestration rate of 1.52 t CO_2 ha^{-1} yr^{-1} for the accumulation in the top 30 cm soil profile[21], an additional soil carbon sequestration potential of about 25 Mt CO_2 yr^{-1} is estimated from natural vegetation regrowth of the 17.1 Mha of net suboptimal cropland identified. This represents about 36% of the aboveground carbon sequestration potential (70.0 Mt CO_2 yr^{-1}). Different responses to SOC are also reported for afforestation of agricultural soils, with existing meta-analyses showing that SOC in planted forests can either increase, decrease, or remain constant depending on specific factors such as tree species, local climate, soil type, and previous land use[31-33]. Fixed ratios are sometimes used to link biomass and soil carbon changes, but they are reported to overestimate soil carbon

accumulation[33]. Using a global mean factor of SOC changes[34] after afforestation averaged between coniferous and deciduous species of 0.71 t CO_2 ha^{-1} yr^{-1} could increase the carbon sequestration potential of the suboptimal cropland suitable for afforestation (8.30 Mha) of about 5.89 Mt CO_2 yr^{-1}, or 7.22% of the aboveground carbon sequestration potential. Given the lack of robust spatially explicit datasets and the large variability found in individual estimates, we refrained from directly including SOC changes in our analysis. Therefore, the climate benefits of the investigated scenarios can reasonably be expected to be higher, and the present estimates can be interpreted as conservative.

Our analysis of biodiversity effects revealed that natural regrowth or afforestation on suboptimal cropland (**Supplementary Fig. 15**) could enhance local species richness by 79.0±12.8% and 24.8±8.81%. This aligns with previous site-based observations[35], which reported that natural regrowth and afforestation in cropland could increase species richness by 51-78% and 18-41%, respectively (**Supplementary Tab. 9**). Our analysis only addresses transitions between cropland and forests or natural vegetation, neglecting potential impacts of intensification on biodiversity in situ on cropland. The consideration of species density–crop yield relationships show rapidly decreasing species density already at low yields, thereby favoring land sparing as a conservation strategy instead of more wildlife-friendly farming[36,37]. At the same time, the species richness within intensive cropland areas may depend on multiple factors such as crop diversity, nutrient inputs, pesticide applications, and other complex local factors that prevent their assessment in spatially explicit studies at a continental scale.

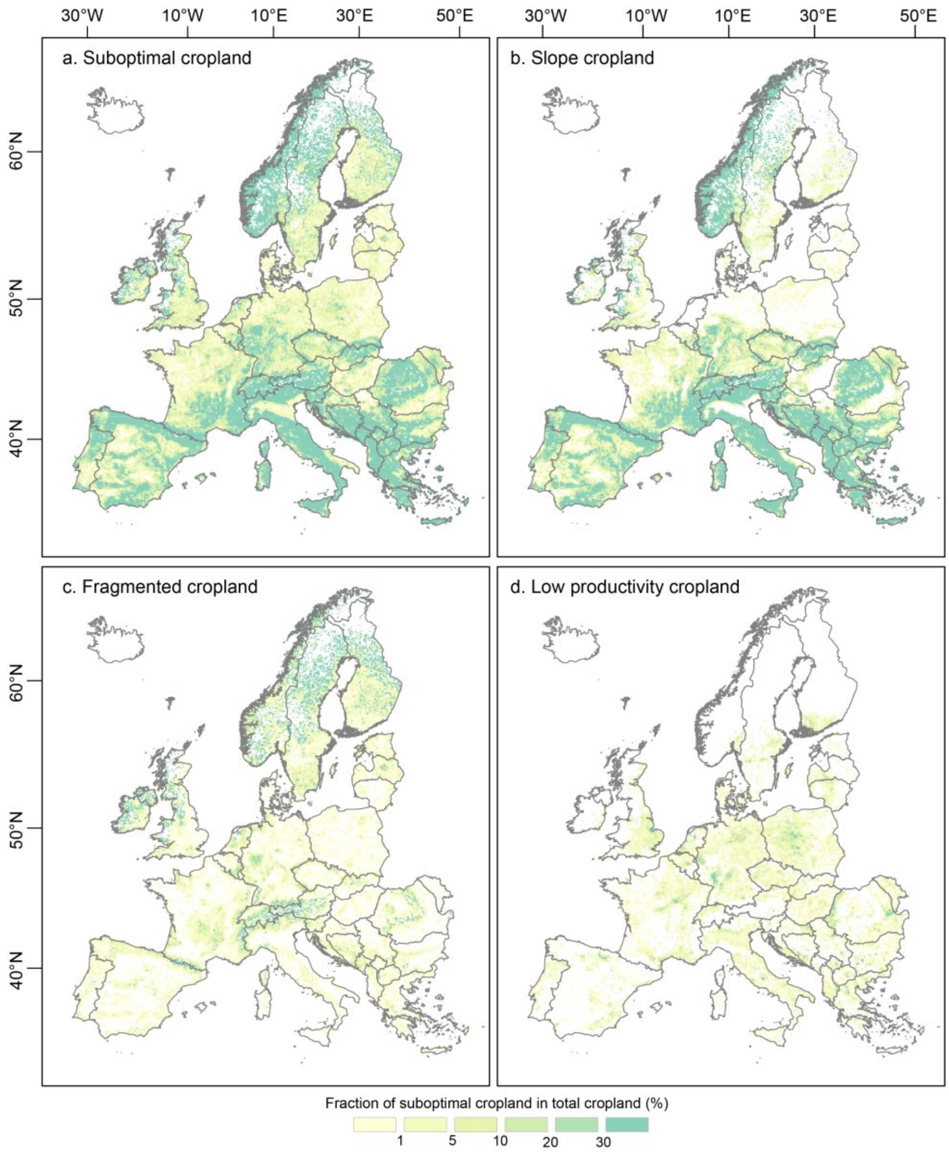

Supplementary Fig. 1 Fraction of suboptimal cropland relative to the total cropland area in each pixel. a) suboptimal cropland, b) slope cropland, c) fragmented cropland, and d) low productive cropland. The map has a resolution of about 10 km × 10 km for visualization purposes, but the analysis is conducted at a resolution of 300 m. Basemaps in (a-d) are sourced from GADM (https://gadm.org), with Austrian data licensed under Creative Commons Attribution-ShareAlike 2.0 (source: Government of Austria).

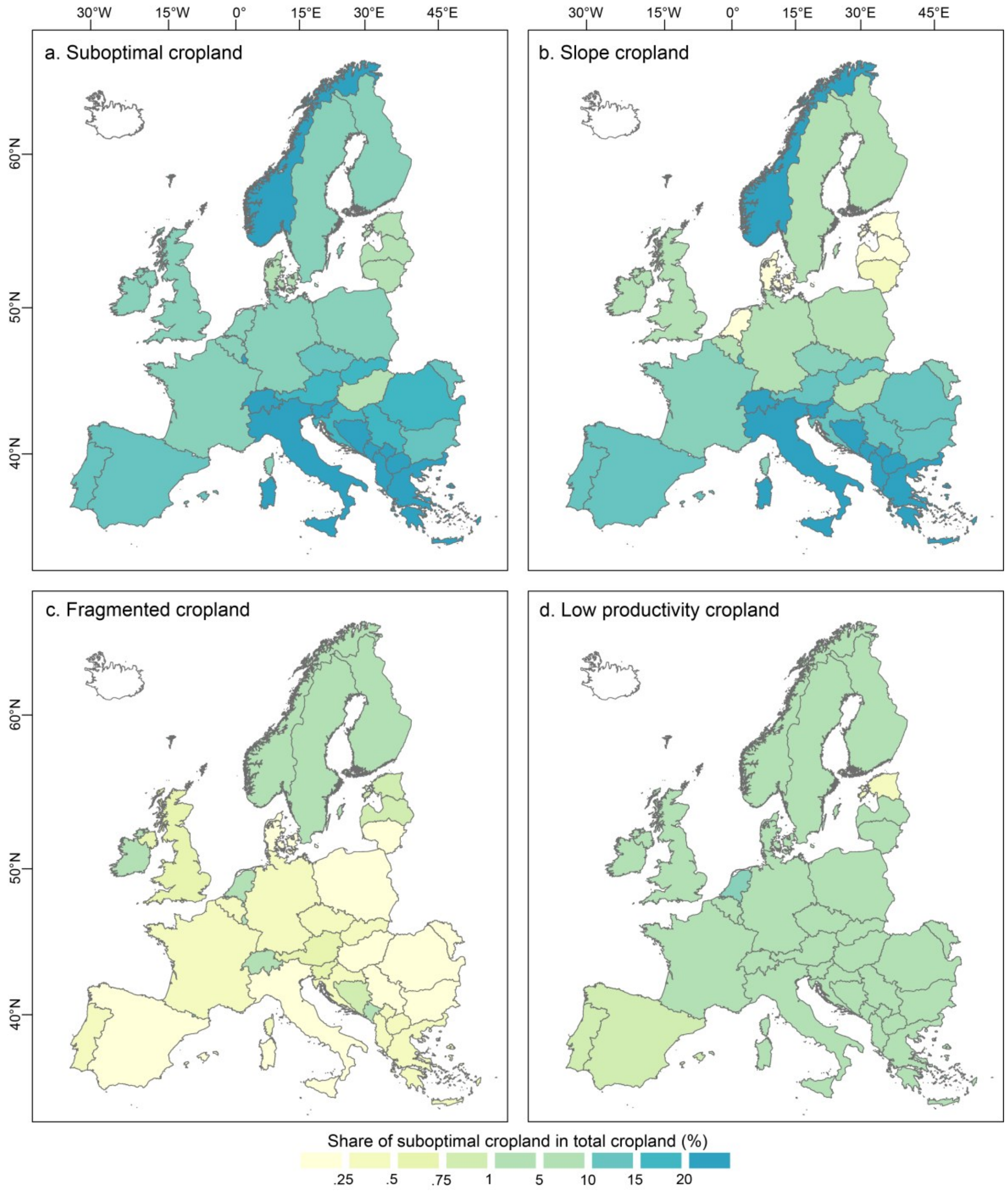

Supplementary Fig. 2 Share of suboptimal cropland relative to the total cropland area in each country. a) suboptimal cropland, b) slope cropland, c) fragmented cropland, and d) low productive cropland. Basemaps in (a-d) are sourced from GADM (https://gadm.org), with Austrian data licensed under Creative Commons Attribution-ShareAlike 2.0 (source: Government of Austria).

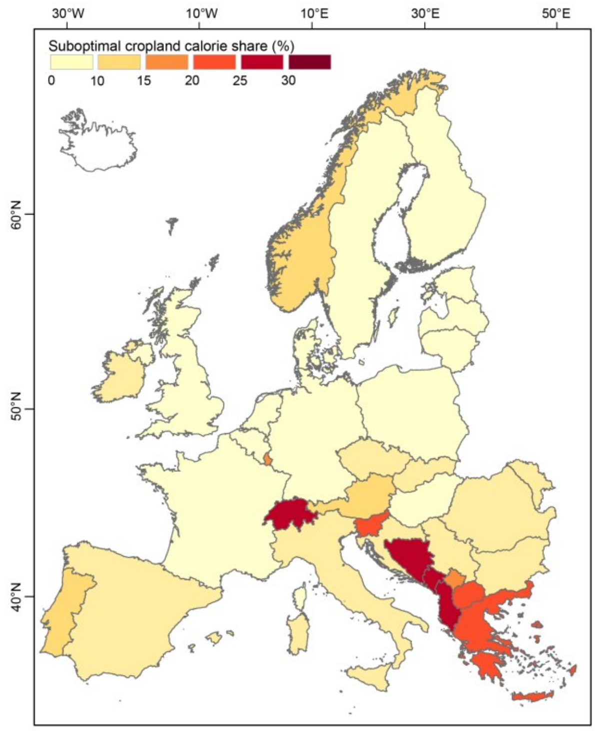

Supplementary Fig. 3 Share of calorie supply from crops grown in suboptimal cropland (%) relative to the total calorie supply from these crops in each country. The estimation of calorie supply is based on cereal crops, oil crops, sugar crops, and root crops. Basemap is sourced from GADM (https://gadm.org), with Austrian data licensed under Creative Commons Attribution-ShareAlike 2.0 (source: Government of Austria).

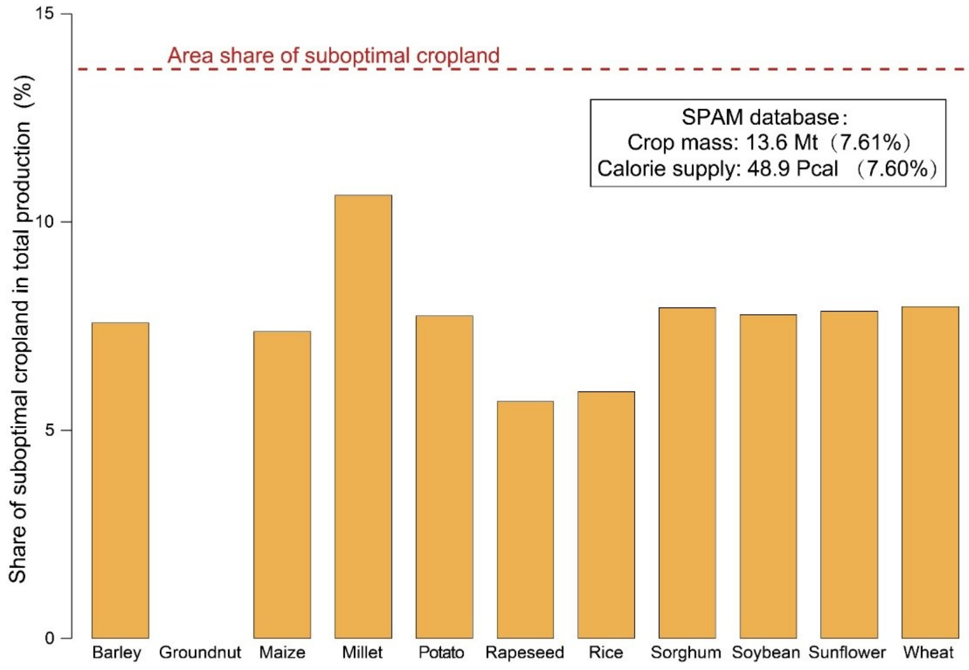

Supplementary Fig. 4 Contribution of suboptimal cropland to crop production according to the Spatial Production Allocation Model (SPAM) database[38]. The Y axis refers to the share of the crop's production in the suboptimal cropland relative to the total crop production in Europe of that crop. The red dashed line indicates the share of suboptimal cropland area relative to total cropland in Europe (13.7%). When the yellow bar is below this line, the productivity of the suboptimal cropland does not match the average yield of that specific crops at European level. The estimation of crop mass considers cereal crops, oil crops, sugar crops and root crops, and the estimation of calorie supply considers cereal crops, oil crops, sugar crops, root crops, vegetables and pulses, consistently with the calculations of **Fig. 2** based on Global Agro-Ecological Zones (GAEZ+2015) [18].

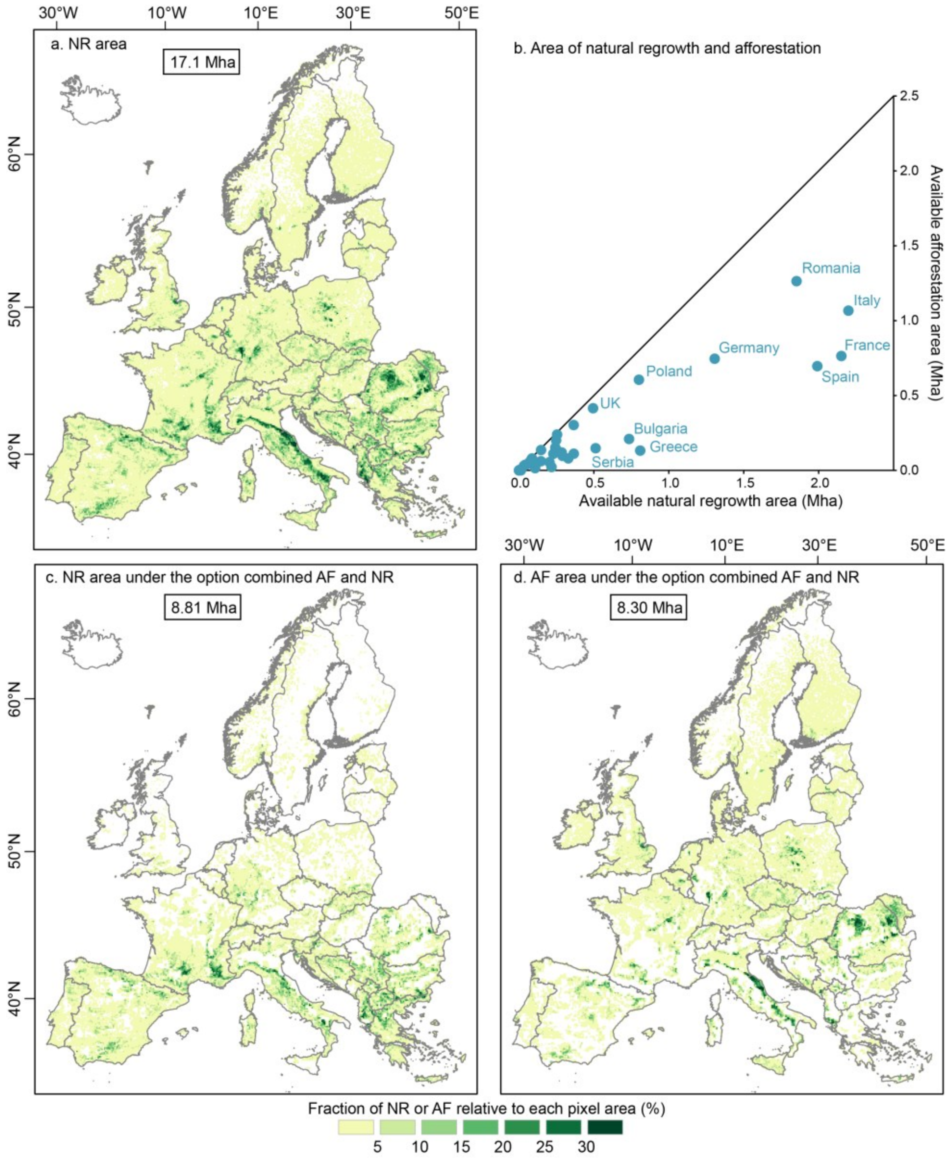

Supplementary Fig. 5 Revegetation options considered for the suboptimal cropland. a) fraction (%) of natural vegetation regrowth (NR) area to each pixel area in the suboptimal cropland, only adopting natural vegetation regrowth while retaining woody perennial crops (e.g., olive, nut tree and fruit trees). b) is the national area (Mha: million hectares) of available natural regrowth or available afforestation (AF) in the suboptimal cropland based on subfigure a) and d). c) and d) show the fraction (%) of natural vegetation regrowth and afforestation when the two options are combined, with afforestation only allowed in regions outside biodiversity priorities and water scarcity. In subfigure a), c) and d), the numbers indicate the corresponding area of natural vegetation regrowth or afforestation. Maps are aggregated to 10 km resolution for better visualization, from an original resolution of about 300 m at which the analysis is performed. Basemaps in (a, c and d) are sourced from GADM (https://gadm.org), with Austrian data licensed under Creative Commons Attribution-ShareAlike 2.0 (source: Government of Austria).

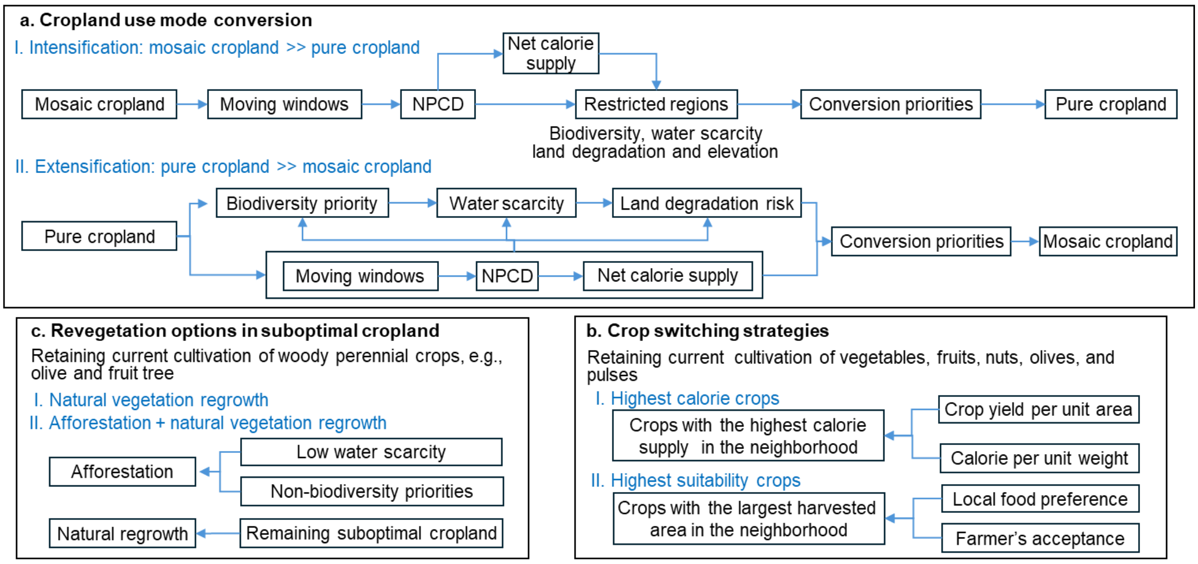

Supplementary Fig. 6 Diagram that shows the main components for the analysis of the 8 scenarios considered in the study: cropland use mode conversion (a), crop switching strategies (b), and revegetation options (c). The combination of two alternatives for each of these three aspects yields a total of eight distinct scenarios (see **Fig. 1**). Cropland use mode conversion includes intensification (i.e., converting mosaic cropland to pure cropland) and extensification (i.e., converting pure cropland to mosaic cropland). For intensification, the conversion is based on the neighboring pure cropland density (NPCD) of existing mosaic cropland, ranked from high to low, excluding the regions of biodiversity priority, land degradation risk, water scarcity and high elevation. For extensification, the conversion prioritizes the area of biodiversity priorities, water scarcity and land degradation risk, ordered by NPCD and net calorie supply before and after crop switching from high to low. Crop switching involves changing currently cultivated crops to that with the highest calorie supply or the ones with the highest local suitability (i.e., crops with the largest harvested areas), while retaining the current cultivation of vegetables, fruits, nuts, pulses, and olives. The woody perennial crops (e.g., olive and fruit trees) in suboptimal cropland are retained, while revegetation of other suboptimal cropland considers natural vegetation regrowth only or afforestation in regions outside biodiversity priorities and water scarcity and with natural regrowth in the remaining suboptimal cropland.

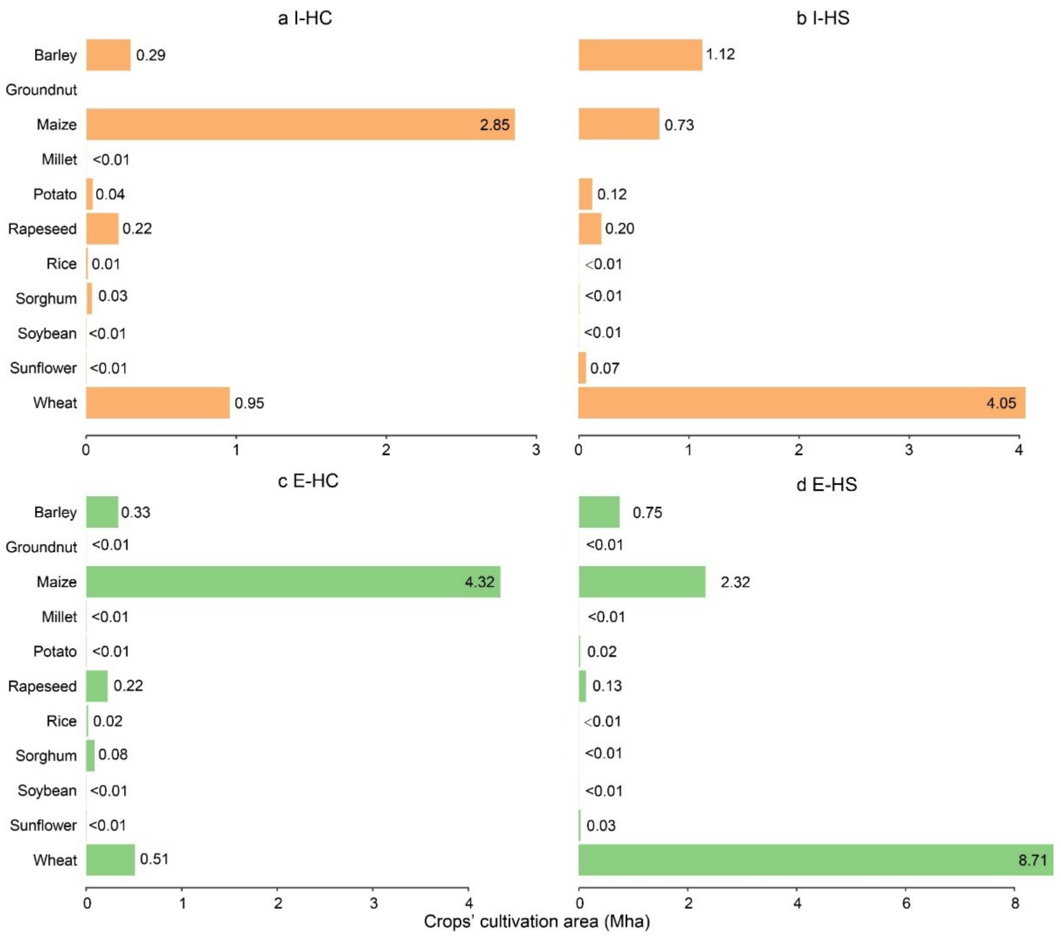

Supplementary Fig. 7 Crop-specific cultivation area in the new pure cropland pixels (intensification) or in the cropland fraction of the new mosaic cropland (extensification) after implementation of crop switching strategies. I: cropland intensification; E: cropland extensification; HC: crops with the highest calorie supply; HS: crops with the highest suitability; Mha: million hectares.

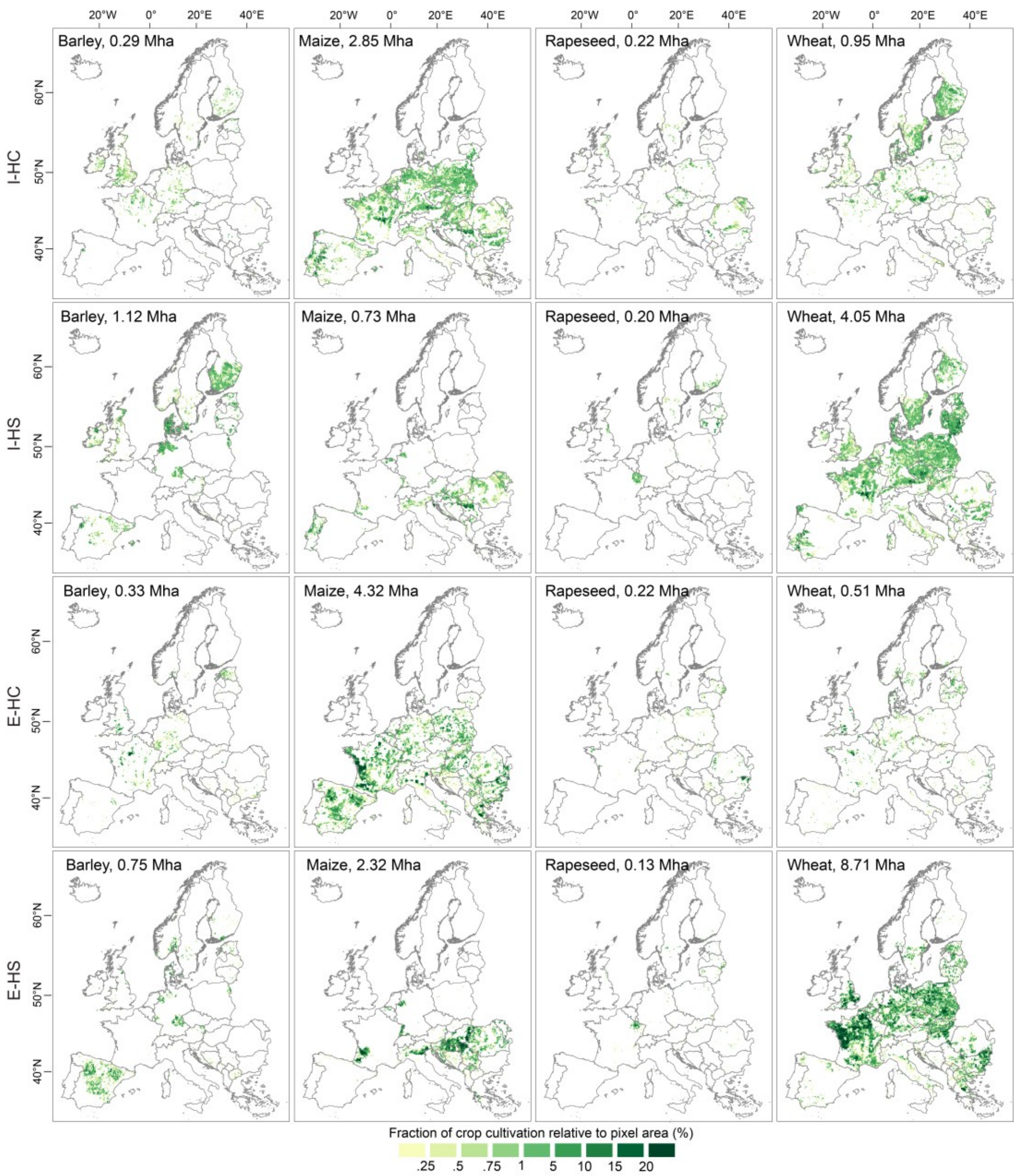

Supplementary Fig. 8 Crop-specific distribution density after crop switching strategies under the different scenarios of intensification and extensification. The color bar refers to cultivation area of each crop in the new pure cropland pixels (intensification) or in the cropland fraction of the new mosaic cropland (extensification), as a percentage of the total pixel area. The number next to the crop name indicates the cultivation area of the crop (Mha: million hectares). Maps are aggregated to a 10 km resolution for better visualization, from an original resolution of about 300 m at which the analysis is performed. Only crops with the top four cultivation areas are listed. I: cropland intensification; E: cropland extensification; HC: crops with the highest calorie supply; HS: crops with the highest suitability. Basemaps are sourced from GADM (https://gadm.org), with Austrian data licensed under Creative Commons Attribution-ShareAlike 2.0 (source: Government of Austria).

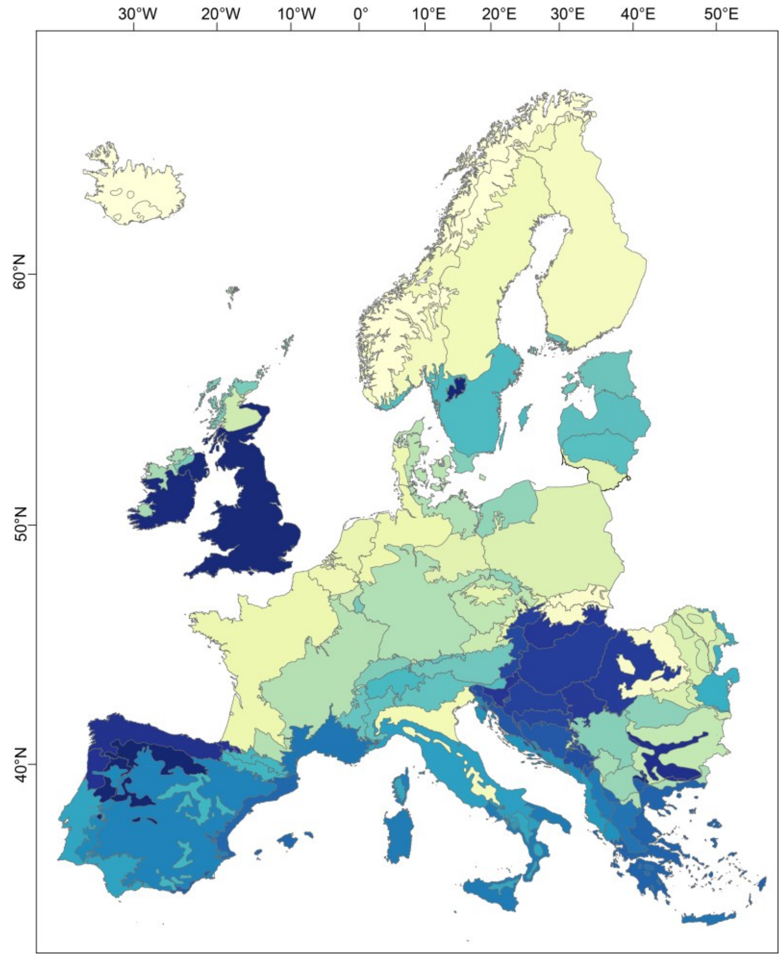

Supplementary Fig. 9 164 subregions in Europe that combine national administrative units and ecoregion units[6]. The different colors are used solely to distinguish the boundaries of subregions for visualization purposes. These subregions are used to identify the distribution of low productive cropland and quantify the impact of cropland extensification on crop yield. Basemap is from GADM (https://gadm.org), with Austrian data licensed under Creative Commons Attribution-ShareAlike 2.0 (source: Government of Austria).

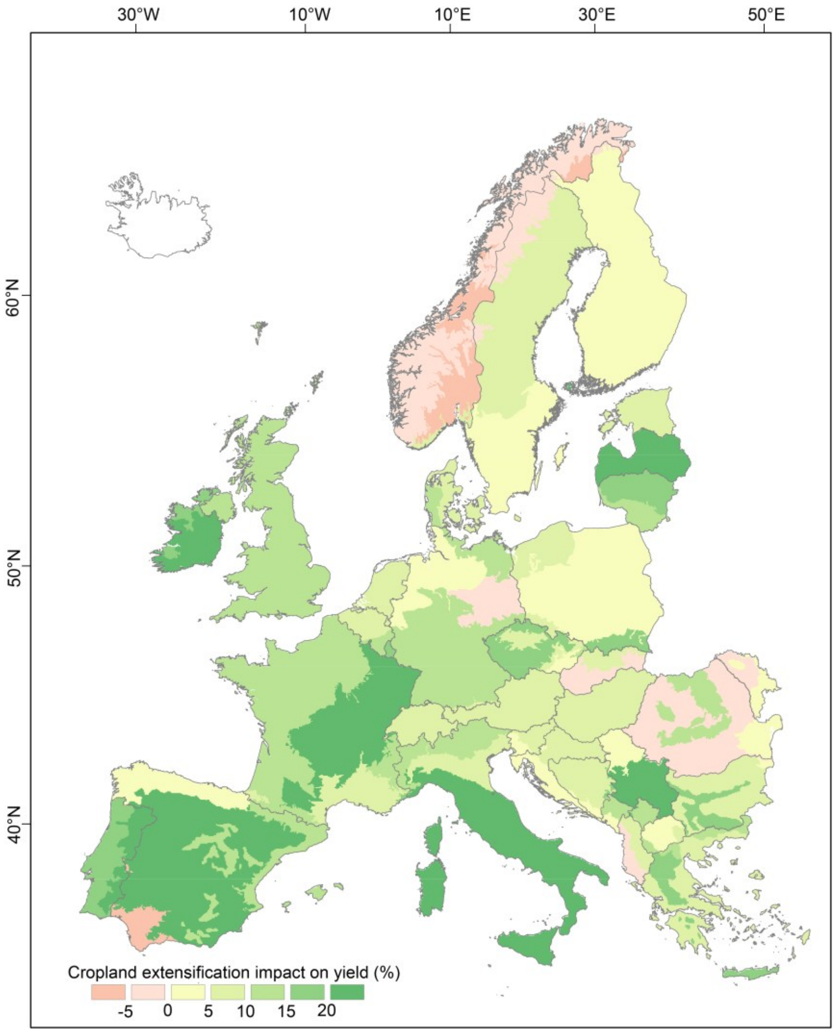

Supplementary Fig. 10 Impact of extensification on crop yields within mosaic cropland pixels (i.e., ω) for 164 subregions that combine national administrative units and ecoregion units. See **Methods and Supplementary Fig. 22** for more details on calculation of impact of extensification on crop yield. Basemap is sourced from GADM (https://gadm.org), with Austrian data licensed under Creative Commons Attribution-ShareAlike 2.0 (source: Government of Austria).

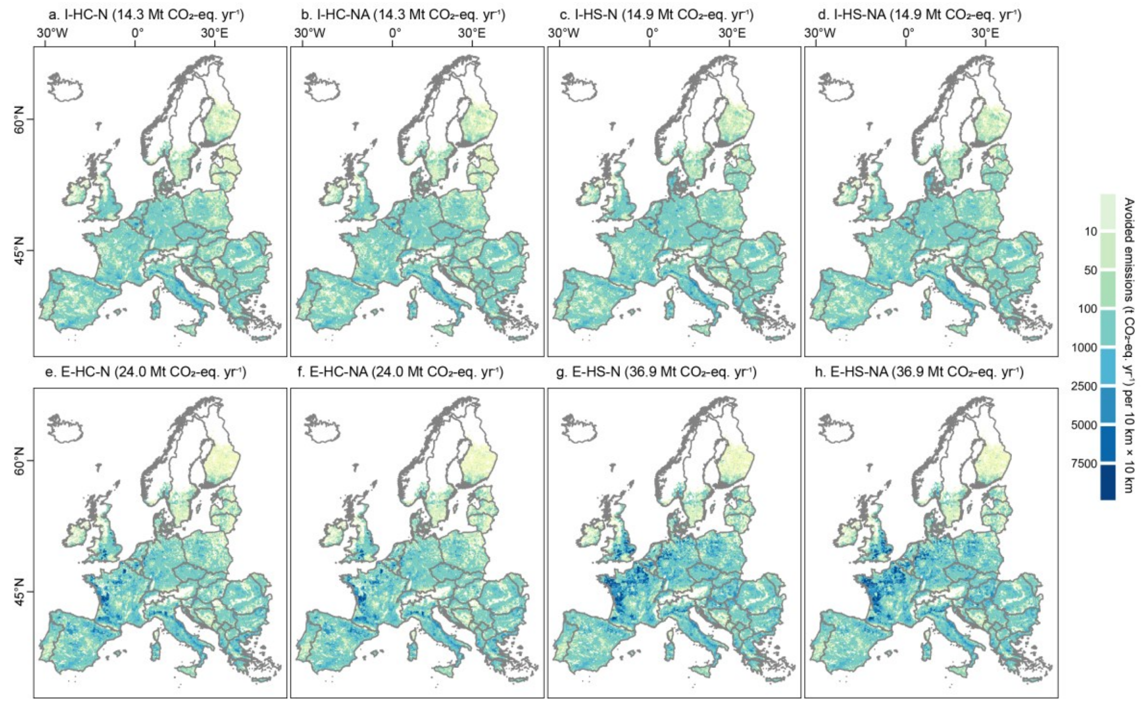

Supplementary Fig. 11 Avoided emissions from fertilization in crop switching and abandoning original crops in suboptimal cropland. I: cropland intensification; E: cropland extensification; HC: crops with the highest calorie supply; HS: crops with the highest suitability; N: natural regrowth; NA: combination of natural regrowth and afforestation. Basemaps in (a-h) are sourced from GADM (https://gadm.org), with Austrian data licensed under Creative Commons Attribution-ShareAlike 2.0 (source: Government of Austria).

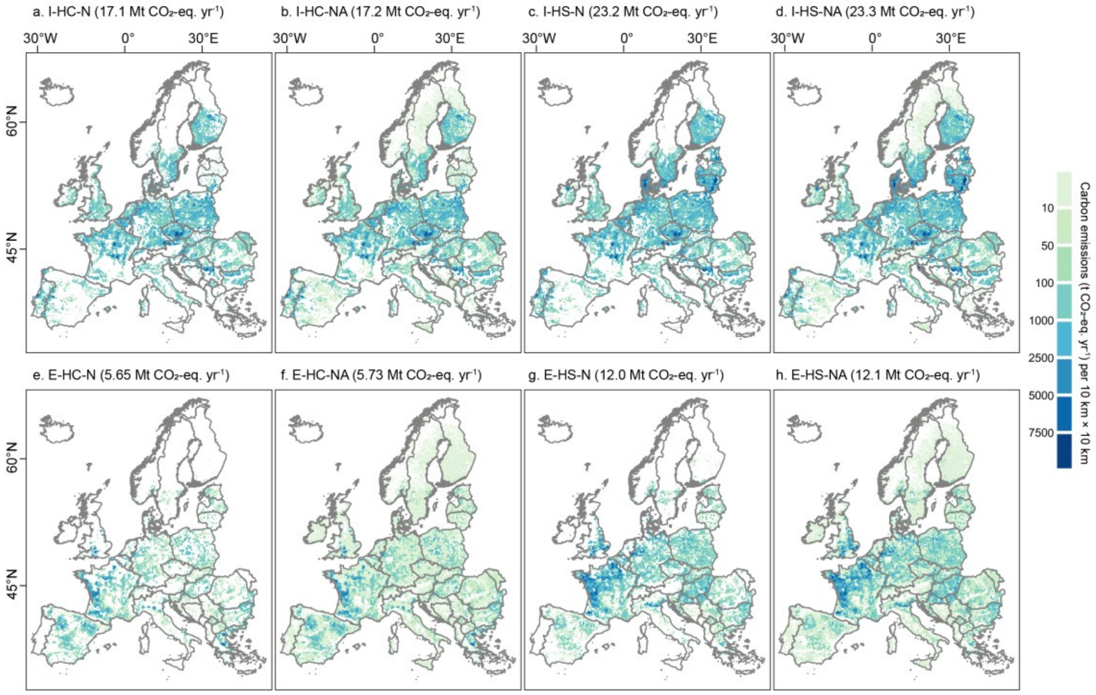

Supplementary Fig. 12 Carbon emissions from clearing aboveground biomass carbon for intensification, emissions from afforestation activities of suboptimal cropland or mosaic cropland, and emissions from fertilization in crop switching. I: cropland intensification; E: cropland extensification; HC: crops with the highest calorie supply; HS: crops with the highest suitability; N: natural regrowth; NA: combination of natural regrowth and afforestation. Basemaps in (a-h) are sourced from GADM (https://gadm.org), with Austrian data licensed under Creative Commons Attribution-ShareAlike 2.0 (source: Government of Austria).

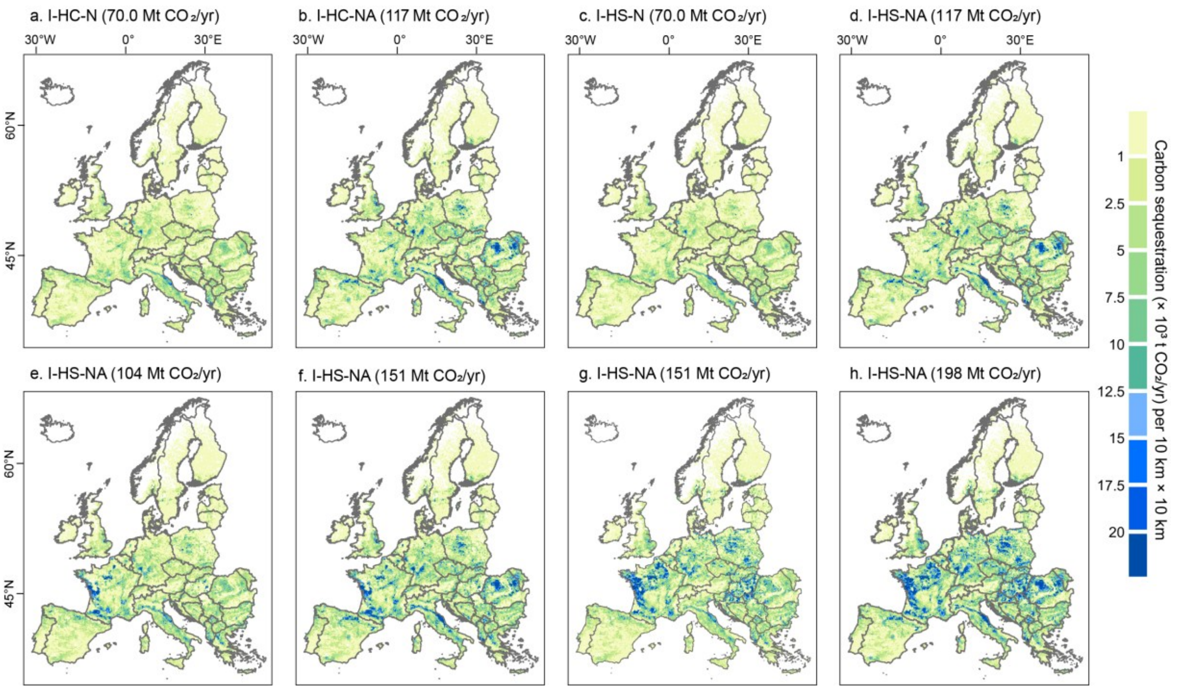

Supplementary Fig. 13 Carbon sequestration from natural vegetation regrowth and afforestation of suboptimal cropland, and trees in new mosaic cropland. I: cropland intensification; E: cropland extensification; HC: crops with the highest calorie supply; HS: crops with the highest suitability; N: natural vegetation regrowth; NA: combination of natural vegetation regrowth and afforestation. Basemaps in (a-h) are sourced from GADM (https://gadm.org), with Austrian data licensed under Creative Commons Attribution-ShareAlike 2.0 (source: Government of Austria).

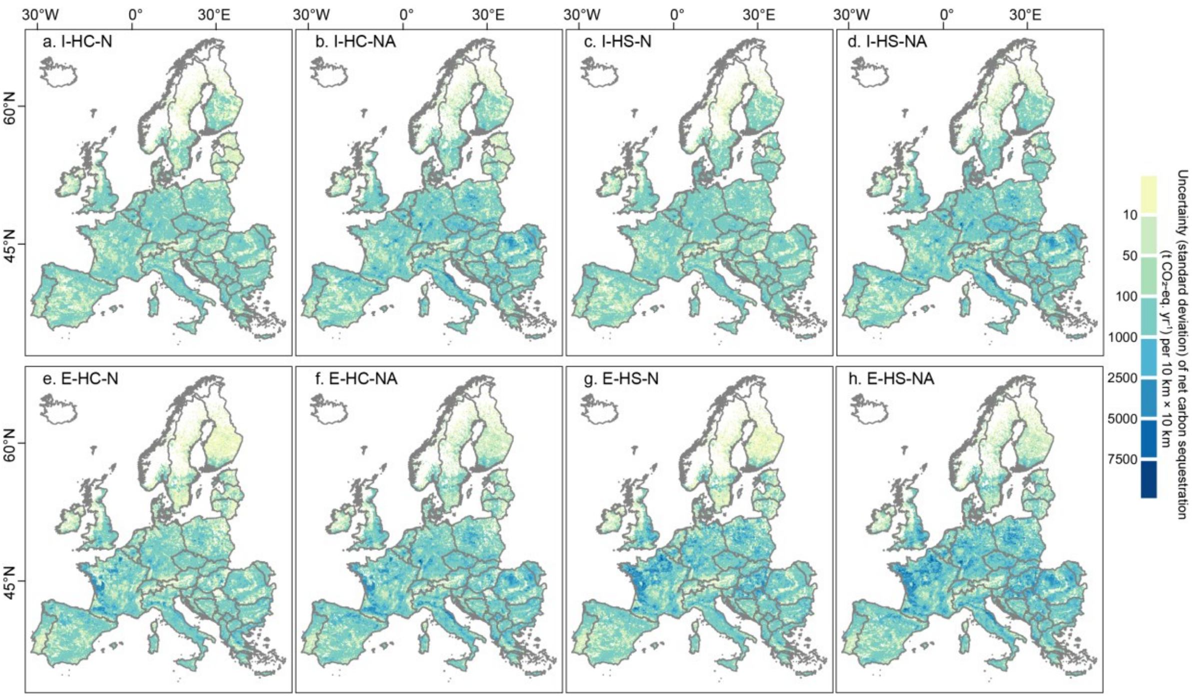

Supplementary Fig. 14 Uncertainty ranges of net carbon sequestration for the eight investigated scenarios that combine cropland use mode (I: cropland intensification, E: cropland extensification) with crop switching strategies (HC: crops with the highest calories supply, HS: crops with highest suitability) and revegetation options (N: natural vegetation regrowth, NA: combination of natural regrowth and afforestation, with afforestation only allowed in regions outside biodiversity priority and water scarcity areas). The uncertainty ranges stem from the datasets used for each source of carbon flow (carbon sequestration from trees in the revegetated suboptimal cropland and in the extensification scenarios, emissions from clearing land and afforestation activities, and fertilization-induced N_2O emissions). Basemaps in (a-h) are sourced from GADM (https://gadm.org), with Austrian data licensed under Creative Commons Attribution-ShareAlike 2.0 (source: Government of Austria).

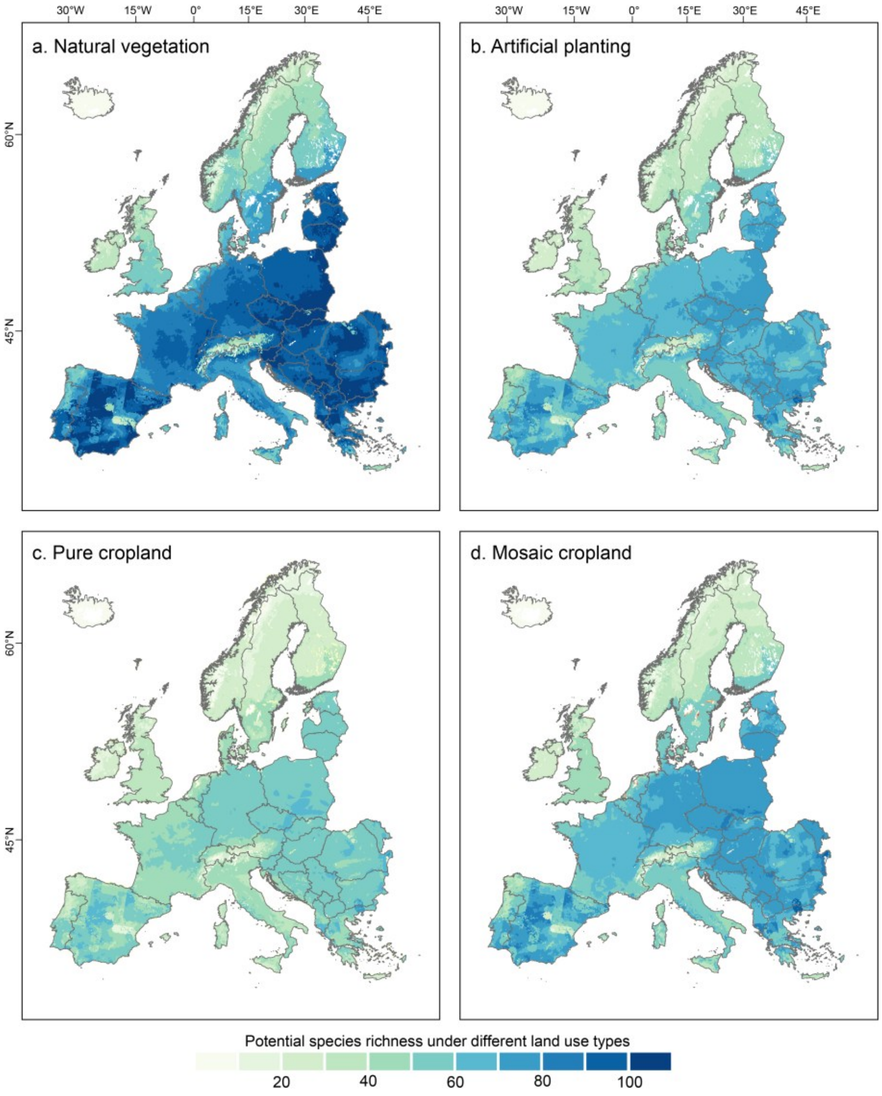

Supplementary Fig. 15 Potential species richness under different land use types. a) natural habitat, b) artificial planting, c) pure cropland and d) mosaic cropland. These maps illustrate the species richness that could be supported if a given area were converted to a specific land use type. Basemaps in (a-d) are sourced from GADM (https://gadm.org), with Austrian data licensed under Creative Commons Attribution-ShareAlike 2.0 (source: Government of Austria).

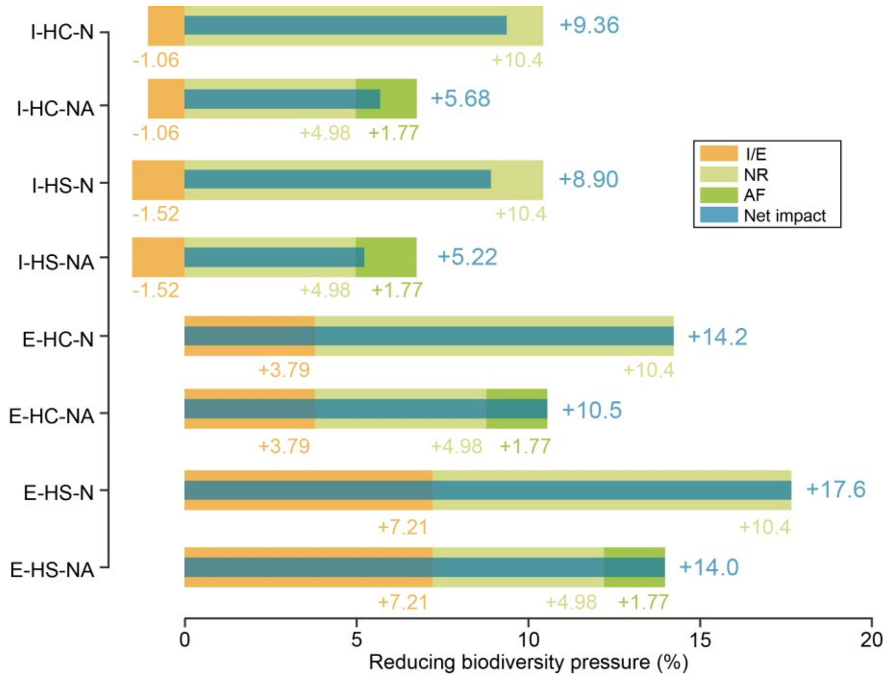

Supplementary Fig. 16 Impact on reduction of biodiversity pressure from the eight investigated scenarios. Bar charts stacked in different colors indicate the net impact on biodiversity (blue), as a contribution of the impact from afforestation (green), natural regrowth (light green) and cropland use mode conversion (yellow). I: cropland intensification; E: cropland extensification; NR: natural regrowth; AF: afforestation. The eight scenarios are indicated on the left side of the figure: HC: crops with the highest calorie supply; HS: crops with the highest suitability; N: natural vegetation regrowth; NA: combination of natural vegetation regrowth and afforestation.

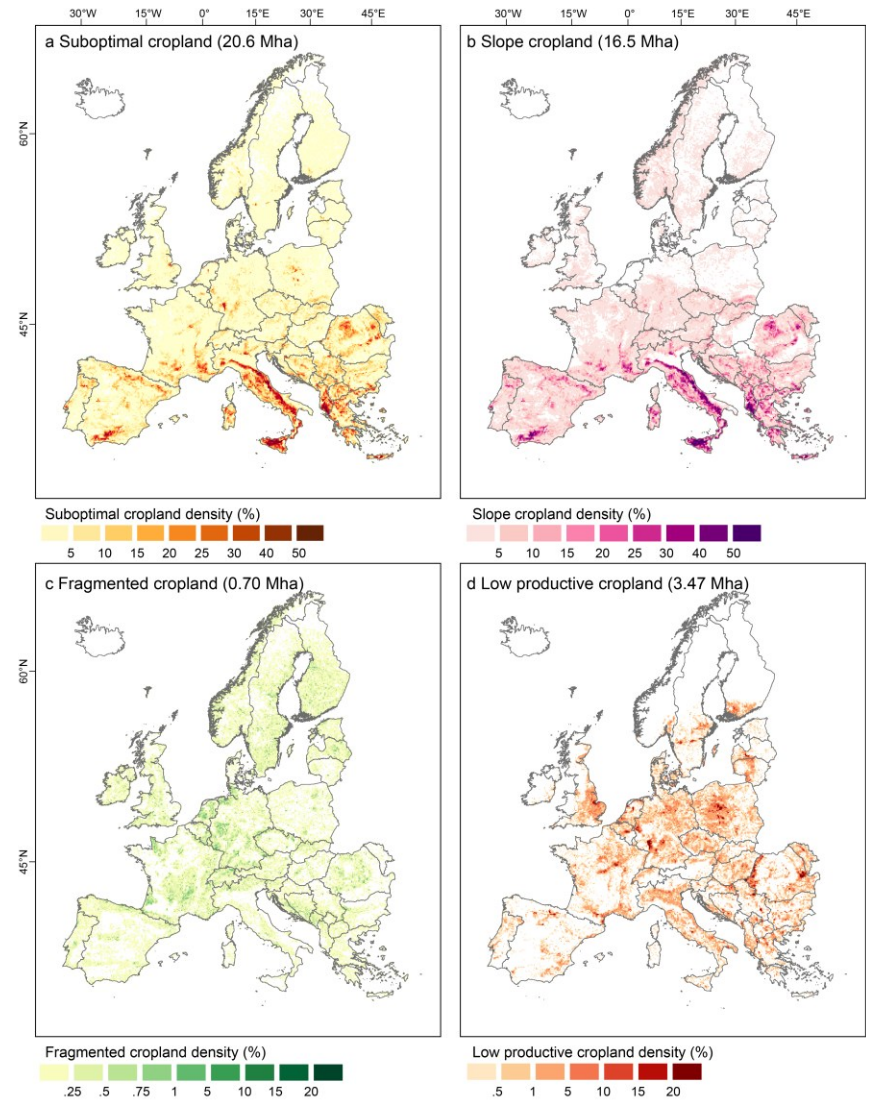

Supplementary Fig. 17 Sensitivity test on suboptimal cropland distribution based on lower-bound estimates. The total identified suboptimal cropland (a) combines cropland in high slope terrains (b), fragmented cropland (c) and low productive cropland (d). Area values are indicated in each panel (Mha: million hectares). Maps are aggregated to 10 km resolution for better visualization, from the original resolution of about 300 m. Threshold values used for the lower-bound estimate are described in the Methods section and summarized in **Supplementary Tab. 5**. Basemaps in (a-d) are sourced from GADM (https://gadm.org), with Austrian data licensed under Creative Commons Attribution-ShareAlike 2.0 (source: Government of Austria).

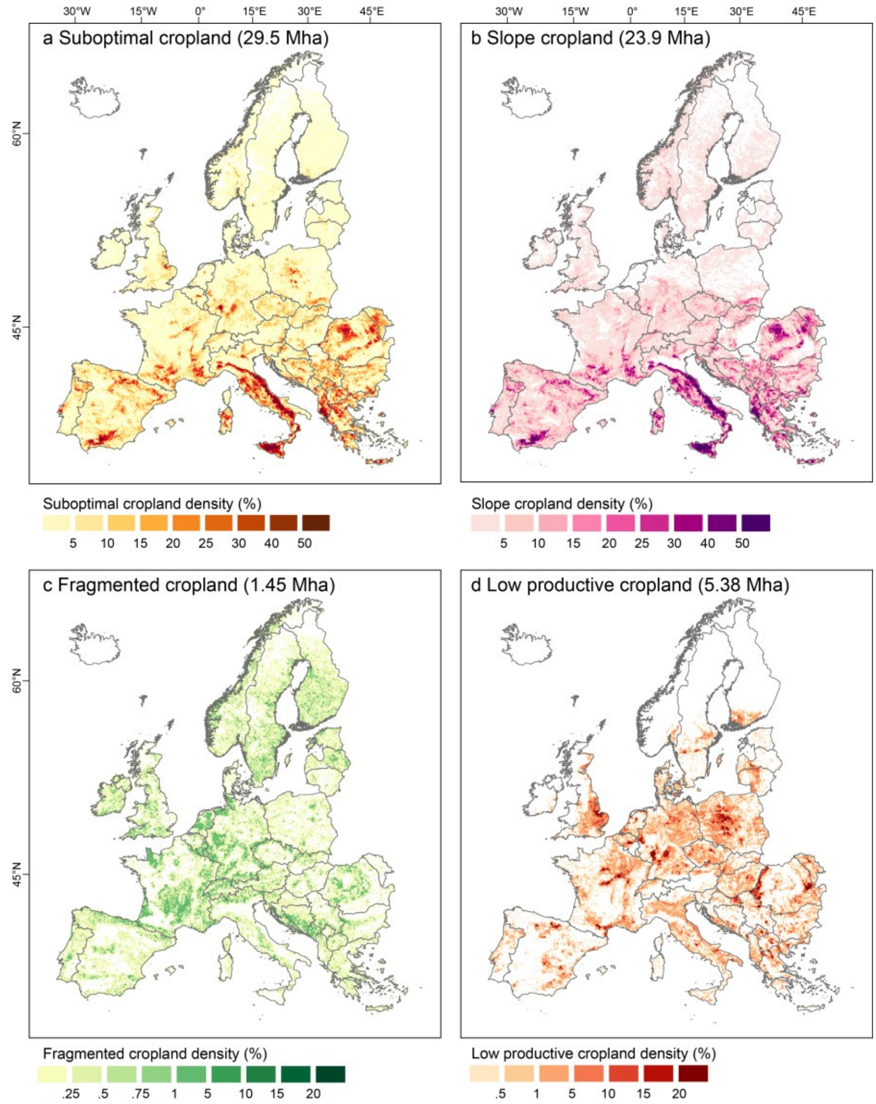

Supplementary Fig. 18 Sensitivity test on suboptimal cropland distribution based on upper-bound estimates. The total identified suboptimal cropland (a) combines cropland in high slope terrains (b), fragmented cropland (c) and low productive cropland (d). Area values are indicated in each panel (Mha: million hectares). Maps are aggregated to 10 km resolution for better visualization, from the original resolution of about 300 m. Threshold values used for the upper-bound estimate are described in the Methods section and summarized in **Supplementary Tab. 5**. Basemaps in (a-d) are sourced from GADM (https://gadm.org), with Austrian data licensed under Creative Commons Attribution-ShareAlike 2.0 (source: Government of Austria).

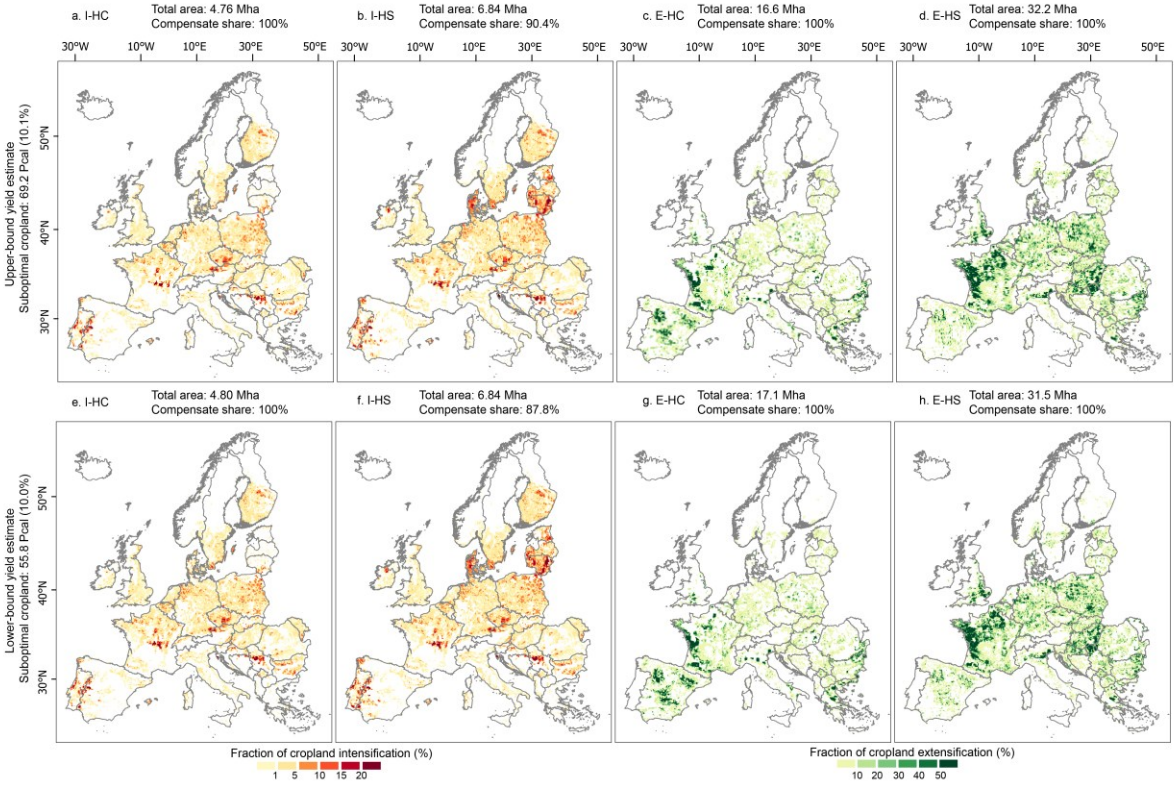

Supplementary Fig. 19 Sensitivity test for crop-specific yield fluctuations on the required area of cropland intensification and extensification. a-d) shows the distribution of cropland under four contrasting approaches under the upper-bound, combining cropland use mode (I: cropland intensification, E: cropland extensification) with crop switching (HC: crops with the highest calories supply, HS: crops with the highest suitability). e-f) shows the distribution of cropland for cropland intensification and extensification under the lower-bound yield. Under the lower-bound yield estimate, suboptimal cropland (excluding woody crops) contributes 55.8 Pcal of food supply, while under the upper-bound estimate, it provides 69.2 Pcal. Maps are aggregated to 10 km resolution for better visualization, from an original resolution of about 300 m at which the analysis is performed. Values in the maps indicate the area requiring intensification or extensification and the compensation share of the calorie losses from revegetating suboptimal cropland. Basemaps in (a-h) are sourced from GADM (https://gadm.org), with Austrian data licensed under Creative Commons Attribution-ShareAlike 2.0 (source: Government of Austria).

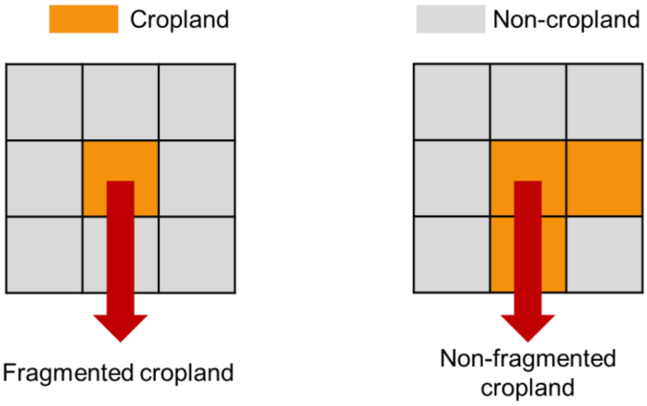

Supplementary Fig. 20 Simplified scheme representing identification of fragmented cropland. Isolated cropland pixels (including pure and mosaic cropland) without nearby cropland (or mosaic cropland) pixels are classified as fragmented.

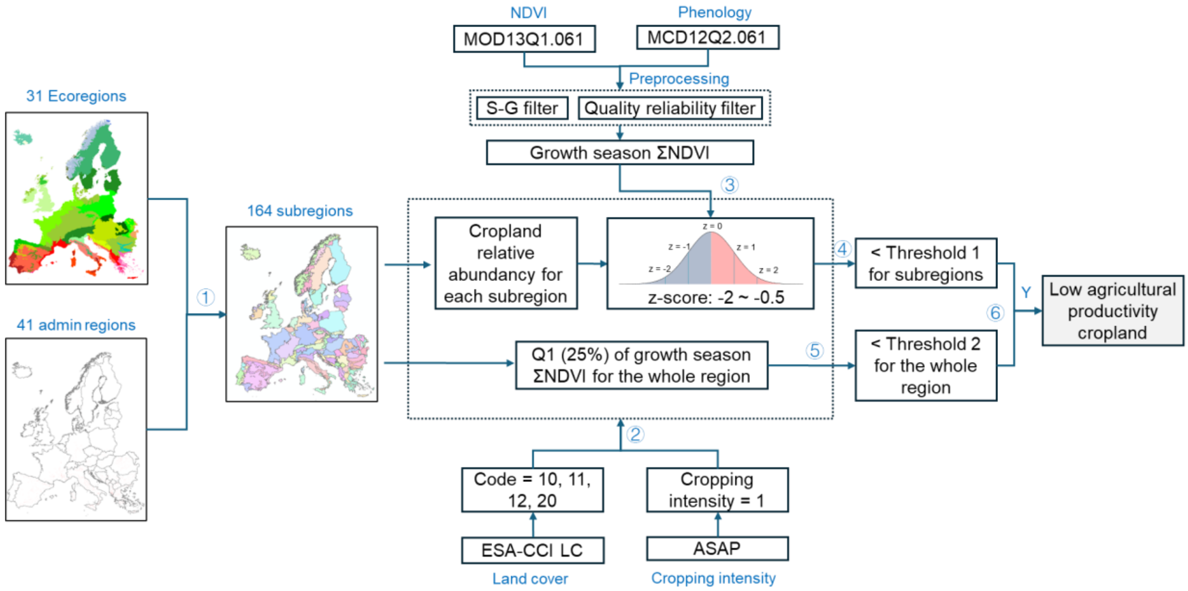

Supplementary Fig. 21 Schematic diagram illustrating the procedure for identification of low productive cropland. The procedure is implemented in 164 subregions that combine 41 national administrative units and 31 ecoregion units[6]. Growing season's cumulative NDVI after Savitzkey-Golay (S-G) filter and quality reliability filter was used to represent agricultural productivity. The identification of low productivity cropland is restricted to pure cropland pixels based on European Space Agency - Climate Change Initiative (ESA-CCI) land cover product[39] with cropping intensity of 1 (i.e., one crop type produced per year) derived from Anomaly Hotspots of Agricultural Production (ASAP) dataset[5]. The z-score method was used to determine the low productivity threshold for each subregion and the lower quartile (Q1: 25%) of the growing season average NDVI of all pure cropland pixels was determined as the threshold for the whole region. The cropland pixels meeting both conditions are classified as low productivity pixels. Basemaps in the methodological flowchart are sourced from GADM (https://gadm.org), with Austrian data licensed under Creative Commons Attribution-ShareAlike 2.0 (source: Government of Austria).

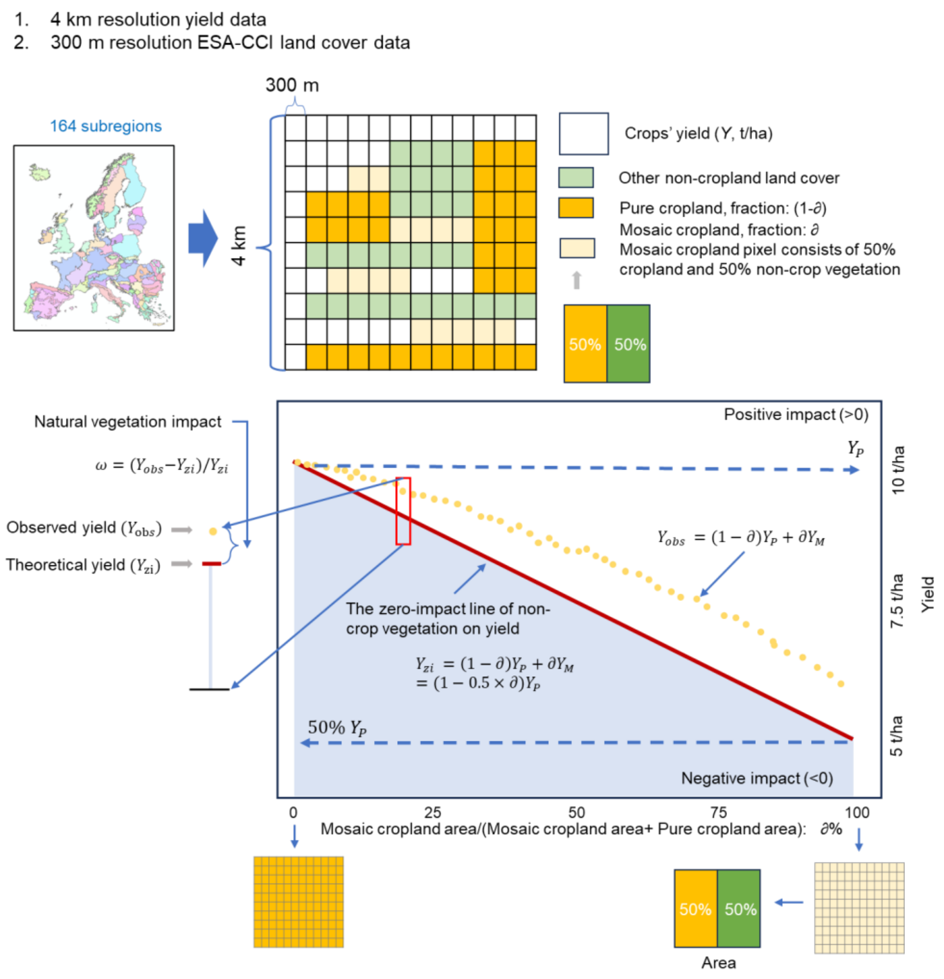

Supplementary Fig. 22 Schematic figure representing the unmixing of the cropland extensification's impact on crop yield across 164 subregions. The upper part of the figure shows the subregions in Europe and the comparison between the grid size of the yield database (4 km)[9] and the land cover dataset (300 m)[39] with the main involved land cover types. In the panel below, the X axis indicates the area proportion of mosaic cropland to total cropland, and the Y axis the yield. The yellow points (i.e., Y_{obs}) indicate the observed yield based on the 4 km yield database. A zero-impact straight line (i.e., red line, Y_{zi}) represents the conditions in which natural vegetation does not affect cropland yields within mosaic cropland, as defined by the yield of fully pure cropland ($\partial = 0$, Y_P) and the yield of mosaic cropland ($\partial = 1$, 50% of the Y_P, assumed that mosaic cropland is composed of 50% cropland and non-crop vegetation). The difference between Y_{obs} and Y_{zi} reflects the impact of non-crop vegetation on cropland yields within mosaic pixels. Any points above the zero-impact line indicate positive impacts, and vice versa. Basemap in the methodological flowchart is sourced from GADM (https://gadm.org), with Austrian data licensed under Creative Commons Attribution-ShareAlike 2.0 (source: Government of Austria).

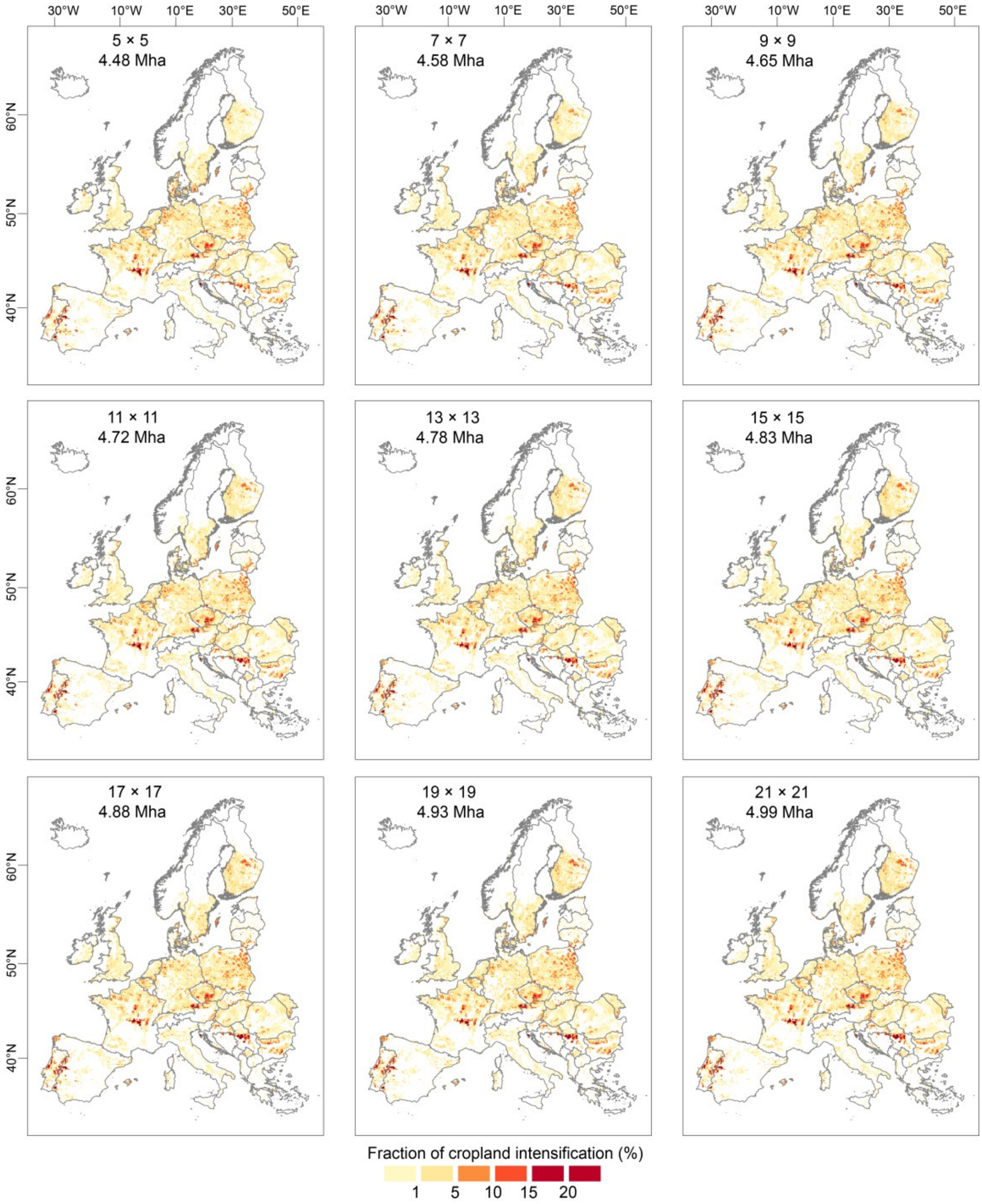

Supplementary Fig. 23 Sensitivity test on moving window's size ranging from 5×5 to 21×21. These figures illustrate the distribution of cropland under intensification and crops switching to highest-calorie crops, using various moving window sizes for cropland density calculations. The resulting area of cropland intensification is indicated for each figure. Maps show the fraction of cropland intensification relative to the pixel area and are aggregated to 10 km resolution for better visualization, from an original resolution of about 300 m at which the analysis is performed. Basemaps are sourced from GADM (https://gadm.org), with Austrian data licensed under Creative Commons Attribution-ShareAlike 2.0 (source: Government of Austria).

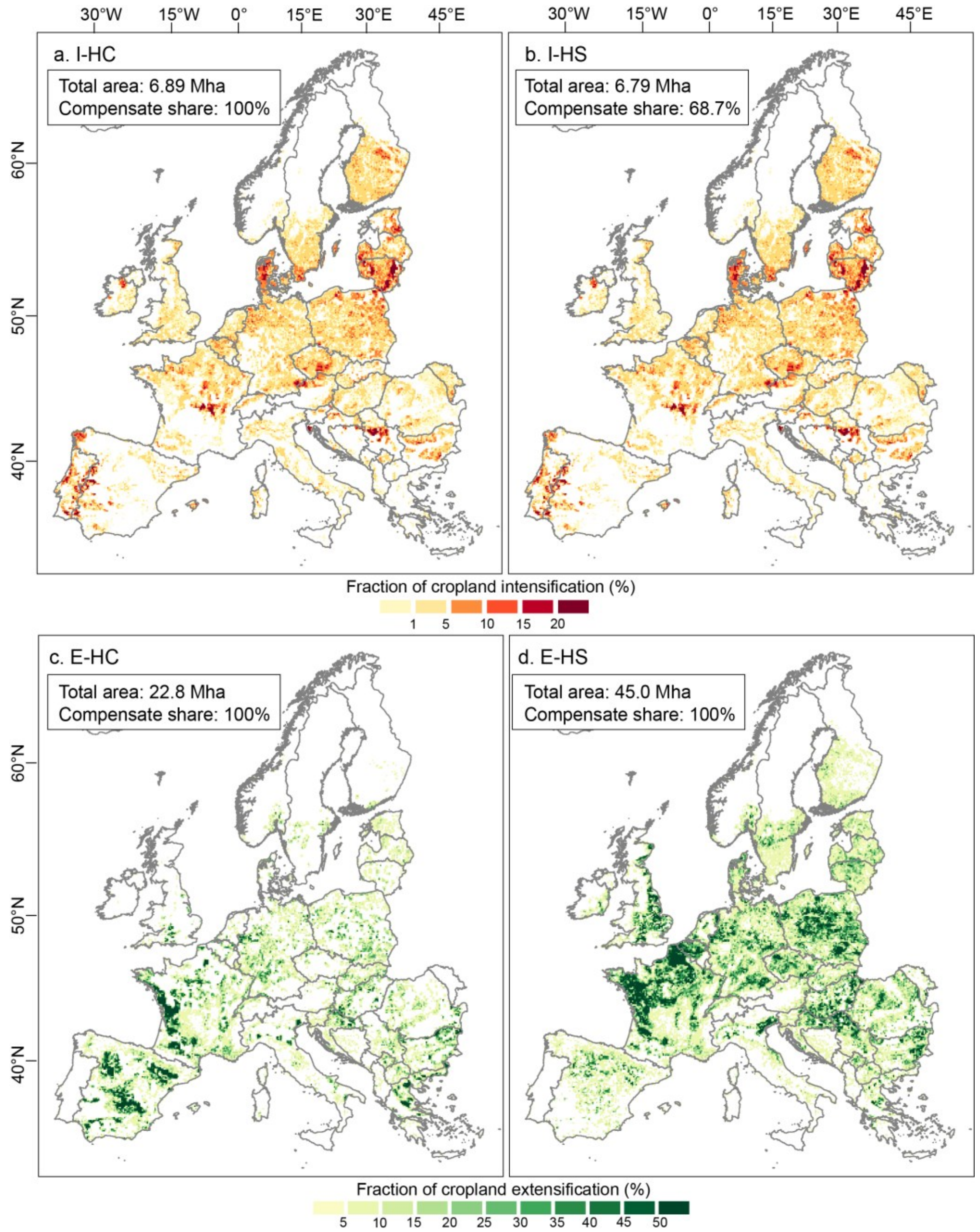

Supplementary Fig. 24 Sensitivity test for the crop harvest rate following crop switching strategies, here considered to be 80% (instead of 100% as in **Fig. 4**). a-d) shows the distribution of cropland under four contrasting approaches combining cropland use mode (I: cropland intensification, E: cropland extensification) with crop switching (HC: crops with the highest calories supply, HS: crops with the highest suitability). Maps are aggregated to 10 km resolution for better visualization, from an original resolution of about 300 m at which the analysis is performed. Values in the maps indicate the area requiring intensification or extensification and the compensation share of the calorie losses from revegetating suboptimal cropland. Basemaps in (a-d) are sourced from GADM (https://gadm.org), with Austrian data licensed under Creative Commons Attribution-ShareAlike 2.0 (source: Government of Austria).

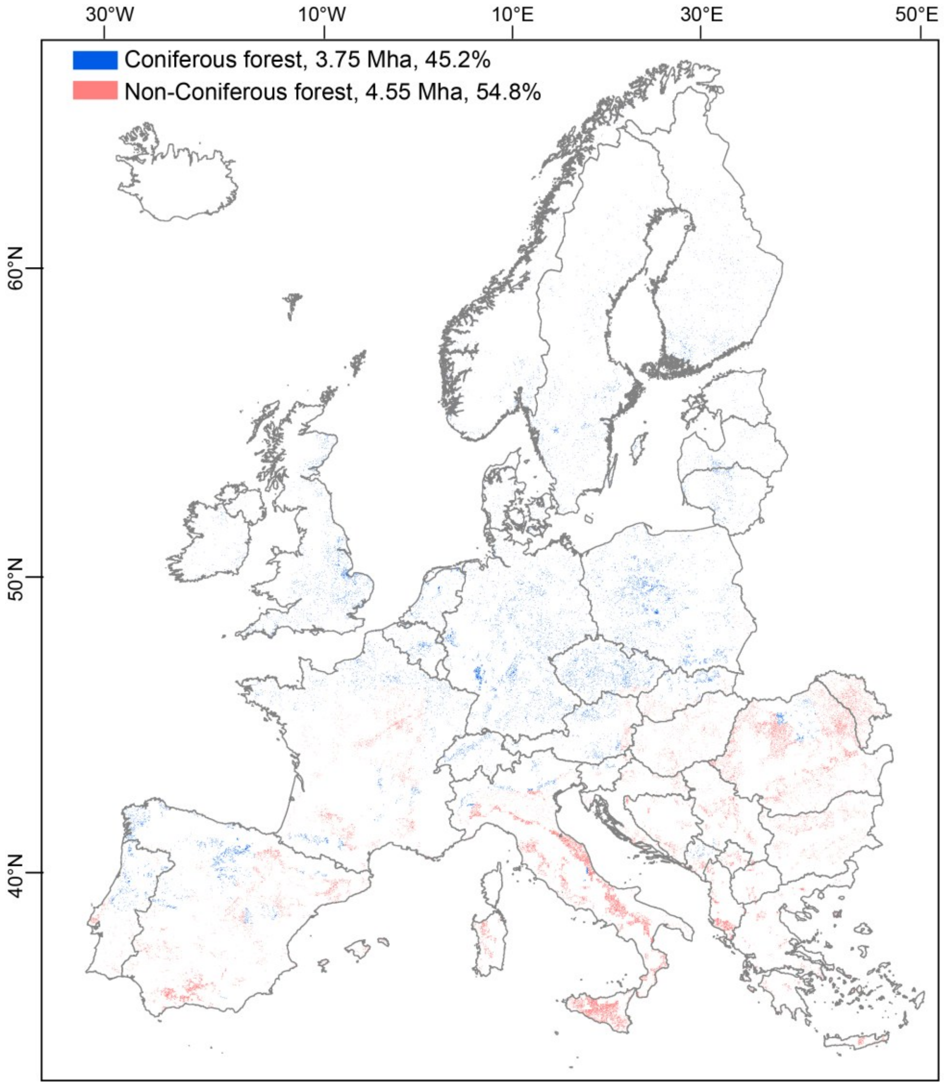

Supplementary Fig. 25 Forest species selection for afforestation of suboptimal cropland. The selection of forest species is according to the maximum carbon sequestration potential per pixel based on G4M model[40] (coniferous forest: blue, non-coniferous forest: red). At a European level, the resulting distribution of tree species on suboptimal cropland is 45.2% coniferous (3.75 Mha) and 54.8% non-coniferous (4.55 Mha). Non-coniferous species are mostly located in the southern part of the domain, while the other species are more common in colder climates. Maps are aggregated to 10 km resolution for better visualization, from an original resolution of about 300 m at which the analysis is performed. Basemap is sourced from GADM (https://gadm.org), with Austrian data licensed under Creative Commons Attribution-ShareAlike 2.0 (source: Government of Austria).

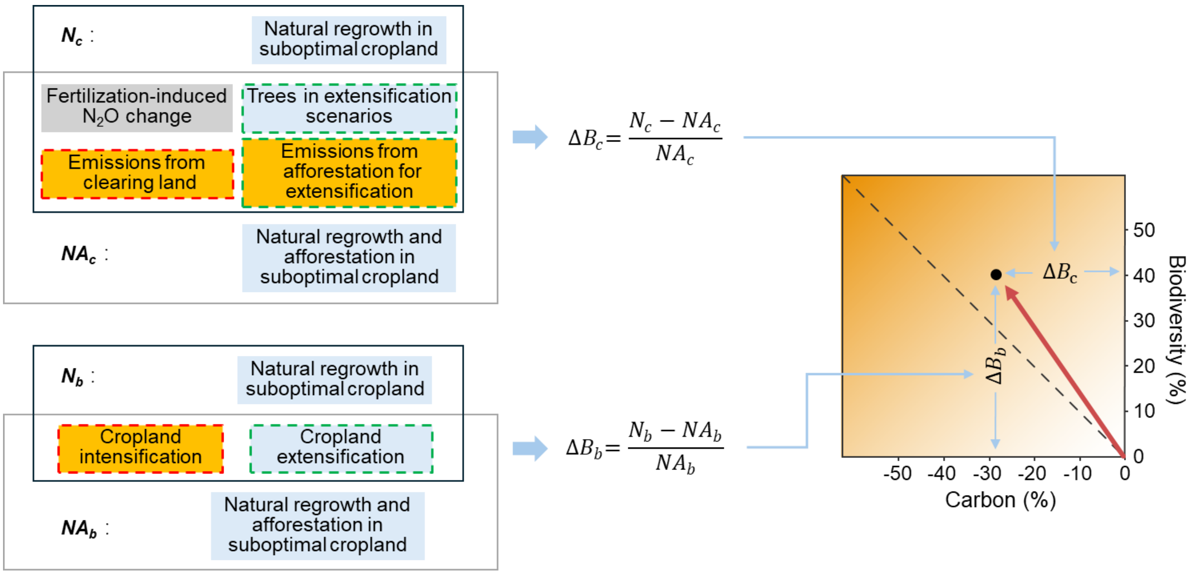

Supplementary Fig. 26 Schematic diagram illustrating the comparison of carbon and biodiversity benefits across different land-use scenarios, which share the same cropland use mode conversion and crop switching strategies, except for differences in revegetation options applied to suboptimal cropland. The analysis compares the total carbon (via climate change mitigation) or biodiversity (via reducing biodiversity pressure from cropland) benefits of natural regrowth and intensification (or extensification) against a combination of natural regrowth, afforestation and agricultural intensification (or extensification), such as E-HC-N versus E-HC-NA. Left panel refers to processes considered in the calculation of N_c, NA_c, N_b, and NA_b. N_c and N_b represent the total carbon and biodiversity benefits, respectively, of scenarios relying solely on natural regrowth in suboptimal cropland, while NA_c and NA_b represent the corresponding benefits of scenarios combining natural regrowth and afforestation. Green-framed boxes represent processes related to cropland extensification, while red-framed boxes indicate processes related to cropland intensification. Blue boxes denote expected positive impacts on carbon sequestration or biodiversity conservation; orange boxes denote negative impacts; and grey boxes represent mixed impacts. Middle panel illustrates the calculation equation for ΔB_b and ΔB_c, which are the difference in carbon and biodiversity benefits between two scenarios where cropland use mode and crop switching strategies are consistent while revegetation options differ.

Supplementary Tab. 1 Impact on crop yields of agricultural extensification in different studies. Effects are essentially due to the presence of woody vegetation near cropland.

Impact on yield (%)	Study area	Reference
17.3 (-4.95 ~ 39.5, 95% CI)	365 comparisons of 53 publications in Europe	[41]
0 ~ 40	21 landscape-scale observation sites (4 km × 4 km) in Spain, France and Netherlands	[42]
-5 ~ 30	14 representative agroforestry practices in Switzerland	[43]
30 ~ 60	45 ha of alley cropping (silvo-arable) agroforestry systems in France	[44]
14 ~ 34	Alley cropping system with food and fodder crops in Denmark	[45]
16	Short rotation alley cropping system in Germany	[46]
7 (median)	1215 comparisons from 95 publications on the global scale	[47]
16	Agro-silvo-pastoral parkland of semi-arid western Africa	[48]
20	Agroforestry survey data from 808 households in southern Malawi	[25]

Supplementary Tab. 2 Impact of the investigated crop-switching strategies on the supply of selected protein, macrominerals, and micronutrients, relative to current total supply from cereals, roots and oil crops.

	Protein	Macrominerals				Micronutrients			
		Sodium	Calcium	Magnesium	Potassium	Iron	Zinc	Copper	Selenium
I-HC	-1.81%	18.7%	-8.45%	-5.99%	-2.86%	-3.25%	-3.60%	-4.46%	-5.45%
I-HS	-0.84%	-3.47%	-0.60%	0.26%	-0.96%	-0.25%	0.17%	-1.15%	3.16%
E-HC	-3.83%	36.8%	-16.6%	-12.0%	-5.45%	-6.92%	-7.67%	-8.92%	-12.1%
E-HS	-1.42%	-2.73%	-2.23%	7.49%	-2.36%	0.20%	0.60%	-2.19%	9.27%

I: cropland intensification, E: cropland extensification, HC: crops with the highest calories supply, HS: crops with the highest suitability

Supplementary Tab. 3 Daily intake contribution of cereal, root and oil crops on the supply of selected protein, macrominerals, and micronutrients

	Protein*	Macrominerals**				Micronutrients**			
		Sodium	Calcium	Magnesium	Potassium	Iron	Zinc	Copper	Selenium
Female	28.3%	32.9%	3.26%	31.4%	16.1%	43.4%	26.4%	35.4%	31.3%
Male		29.7%	3.77%	36.6%	18.8%	45.9%	25.1%	40.7%	30.8%

* It is based on average protein intake data from cereal, root and oil crops in European countries, as reported in FAOSTAT[49] for the year 2020.

** It is derived from dietary investigation of 579 healthy participants (274 females and 305 males) in Europe[50].

Supplementary Tab. 4 Overview of country-level policies for supporting revegetation of suboptimal cropland or cropland extensification.

Country/region	Policy or program	Main measures or purpose
UK	Landscape Recovery Scheme[51]	Large-scale rewilding and habitat restoration scheme to restore native habitats, re-wet peatlands, create woodlands, and reconnect rivers on less productive farmland
	Agriculture Act (2020) & Environmental Land Management (ELM) Schemes[52]	The UK's post-CAP framework compensates farmers for delivering environmental services, incentivizing extensive land-management practices, agroforestry systems (e.g., silvoarable), and the establishment of habitat buffer strips.
Germany	Framework Plan of the Joint Task 'Improvement of Agricultural Structure and Coastal Protection' (GAK) 2024–2027[53]	The scheme supports planting mixed woodland—at least 30 % native broad-leaf species—on farmland taken out of production or left fallow, covering up to 100 % of the afforestation costs.
	Aktionsprogramm Natürlicher Klimaschutz[54]	It promotes nature-based solutions to enhance carbon stock and biodiversity conservation in agricultural landscapes: creating or restoring field hedgerows, and adopting agroforestry combining timber, fruit or nut trees with crops
France	National Low Carbon Strategy[55]	Supports both active tree planting and passive natural regrowth in abandoned agricultural land and degraded land.
	Label Bas-Carbone "Méthode Haies"[56]	The program enables farms to earn verified carbon credits by planting or renovating hedgerows under a 15-year management plan. Each plan must include a comprehensive bocage inventory, incorporate a diversity of native species, and prohibit chemical buffer strips
Poland	National Programme for Increasing the Forest Cover (KPZL)[57]	It aims to increase Poland's forest cover to 30% by 2020 and 33% after 2050. In practice, marginal and low-quality agricultural land are key target areas for afforestation.
Italy	Strategia Forestale Nazionale (SFN)[58]	A portfolio of actions to increase forest cover and quality and combat desertification promotes afforestation of marginal

		agricultural lands and degraded land, natural regeneration on slope cropland; and to implement technical guidelines, incentive mechanisms, monitoring protocols, and training programs to support the management of agrosilvopastoral and agroforestry systems
Spain	Plan Forestal Español 2022-2032, PFE[59]	It promotes agrosilvopastoral systems as a vital strategy for conserving and enhancing forest ecosystems, biodiversity, and socio-economic value
Denmark	Aftale om et Grønt Danmark[60]	A broad land-use reform deal to retire a portion of Denmark's least sustainable farmland and convert it to forests or natural habitats. About 250,000 ha of cropland will be afforested over 20 years
Netherlands	"Food-Forest" Green Deal[61]	A food forest is defined as a minimum of 0.5 ha featuring at least three vertical vegetation layers and designed for long-term edible yields. Such projects integrate rows or clusters of fruit, nut, and timber trees within existing cropland, often arranged in alley-cropping layouts.
Romania	PNRR State-Aid scheme (OUG 35/2022)[62]	It facilitates on-farm afforestation and the establishment of windbreaks to reduce wind erosion and enhance water retention in drought-prone plains
Scotland	Forestry Grant Scheme "Agroforestry" Option[63]	It provides capital grants and annual maintenance payments for integrating up to 400 trees per hectare (including biodiversity and fruit/nut species) within pasture or arable land
Ireland	Native Tree Area Scheme[64]	It supports the creation of small native mixed native woodland (oak, birch, rowan, etc.) on farmed land with area < 1 ha.

Supplementary Tab. 5 Thresholds used for suboptimal cropland identification in a sensitivity analysis and corresponding suboptimal cropland area for the high, mean, and low estimate.

	Slope cropland	Fragmented cropland	Low productivity cropland	Total area
Upper-bound estimation	slope >= 7° (23.9 Mha)	isolated cropland patch <= 20 ha (1.45 Mha)	z score: -0.25~ -2 (5.38 Mha)	29.5 Mha
Main text	slope>= 8° (19.7 Mha)	isolated cropland patch <= 10 ha (0.70 Mha)	z score: -0.5 ~ -2 (4.23 Mha)	24.2 Mha
Lower-bound estimation	slope>= 9° (16.5 Mha)	isolated cropland patch <= 10 ha (0.70 Mha)	z score: -0.75~ -2 (3.47 Mha)	20.6 Mha

Supplementary Tab. 6 Review of previous estimates of suboptimal cropland or cropland under some form of degradation in Europe or the globe.

Spatial scale	Year	Area (Mha)	Description	Reference
EU27+UK	2016	43	Cropland with soil erosion risk by water, wind, tillage and harvesting	[10]
Europe	2020	51.35	Marginal agricultural areas (including pasture and cropland) are subject to by biophysical (climate and soil) constraints that make conventional farming challenging and have a higher chance to become abandoned or degraded.	[65]
EU27	2016	29	Agricultural areas (excluding pasture and natural grassland) with moderate (5-10 t/ha/y) or high (>10 t/ha/y) soil erosion rate	[66]
Europe	2001-2012	20	Cropland with low cropping frequencies, i.e., only 1 to 4 years of farming in 12 years	[67]
Europe		37.2	Cropland is currently threatened by medium and high degradation risk	[68]
Globe	2019	100	Steep-slope cropland	[11]

Supplementary Tab. 7 Average carbon sequestration rates of natural vegetation regrowth reported in different studies.

Carbon sequestration ($t\ CO_2\ ha^{-1}\ yr^{-1}$)	Study area	Reference
13.13	Globe	[69]
11.59	Globe	[21]
4.5	Europe	[70]
3.12	Europe	[71]
2.57	European Russia, Ukraine, and Belarus	[72]
4.25-4.55	China	[73]
4.15	Maryland, US	[74]
5.50	Wyoming, US	[75]

Supplementary Tab. 8 Average carbon sequestration rates of afforestation reported in different studies.

Carbon sequestration ($t\ CO_2\ ha^{-1}\ yr^{-1}$)	Study area	Reference
7.7	Globe	[76]
10.34	Global temperate regions	[69]
6.45-8.38	China	[73]
7.7-9.17	Bavaria, Germany	[77]
6.97-8.43	Finland	[78]
11.12	UK	[79]

Supplementary Tab. 9 Impact of natural regrowth and afforestation on species richness in different studies.

Spatial scale	Estimation	Description	Reference
Globe	+18.1%	Species richness in lightly managed plantation forests was 18.1 % higher than in cropland	80
Sichuan, China	+25-41%	Species richness in mixed-forest afforestation areas was 25–41% higher than in adjacent cropland	81
Europe	+35%	Converting cropland into semi-natural habitats increases species richness by 35% based on an analysis of 169 farms in Europe	82
Globe	+67.9%	Natural landscapes support 67.9% higher species richness than croplands embedded in human-modified landscapes	83
Equatorial Andes	+71.7%	Bird species richness is 71.7% higher in natural forests compared to monoculture plantations	84
Globe	+62.7%-77.7%	62.7% of birds and 77.7% of mammal species gain habitat following cropland abandonment, based on site-based observation for 1322 bird and mammal species	35
West Siberia	+51%	Based on surveys of 81 bird species, cropland abandonment increases bird species richness by 51%	85
Mediterranean	+54.8%	Species richness in primary vegetation is 54.8% higher than in harvested agricultural land	86

Supplementary Tab. 10 Definition of steep slopes for agriculture considered in different studies.

Definition of steep slope cropland	Study area	Reference
>10°	Globe	[11]
>12% (i.e., >6.8°)	Globe	[87]
≥7°	Lithuania	[88]
>20% (i.e., >11.3°)	Central America	[89]
>12% (i.e., >6.8°)	Tropics	[90]
>8% (i.e., >4.6°)	Tropical American	[91]

References

1 Benami, E. *et al.* Uniting remote sensing, crop modelling and economics for agricultural risk management. *Nature Reviews Earth & Environment* **2**, 140–159 (2021).

2 Tanaka, T., Sun, L. X., Becker-Reshef, I., Song, X. P. & Puricelli, E. Satellite forecasting of crop harvest can trigger a cross-hemispheric production response and improve global food security. *Communications Earth & Environment* **4** (2023).

3 Martini, G., Bracci, A., Riches, L. et al. Machine learning can guide food security efforts when primary data are not available. *Nature Food* **3**, 716–728 (2022).

4 Didan, K. MODIS/Terra Vegetation Indices 16-Day L3 Global 250m SIN Grid V061. *NASA EOSDIS Land Processes Distributed Active Archive Center* (2021).

5 Rembold, F. *et al.* ASAP: A new global early warning system to detect anomaly hot spots of agricultural production for food security analysis. *Agr Syst* **168**, 247–257 (2019).

6 Olson, D. M. *et al.* Terrestrial ecoregions of the worlds: A new map of life on Earth. *Bioscience* **51**, 933–938 (2001).

7 Horrocks, C. A., Dungait, J. A. J., Cardenas, L. M. & Heal, K. V. Does extensification lead to enhanced provision of ecosystems services from soils in UK agriculture? *Land Use Policy* **38**, 123–128 (2014). 8 van Grinsven, H. J. M., Erisman, J. W., de Vries, W. & Westhoek, H. Potential of extensification of European agriculture for a more sustainable food system, focusing on nitrogen. *Environ Res Lett* **10** (2015).

9 Zhang, Z., Luo, Y. C., Han, J. C., Xu, J. L. & Tao, F. L. Estimating Global Wheat Yields at 4 km Resolution during 1982-2020 by a Spatiotemporal Transferable Method. *Remote Sens-Basel* **16** (2024).

10 Borrelli, P. *et al.* Policy implications of multiple concurrent soil erosion processes in European farmland. *Nature Sustainability* **6**, 103–+ (2023).

11 Wang, L. *et al.* Socioecological Predicament on Global Steeply Sloped Cropland. *Earths Future* **11** (2023).

12 Smith, L. G. *et al.* Assessing the multidimensional elements of sustainability in European agroforestry systems. *Agr Syst* **197** (2022).

13 Seiter, S., William, R. D. & Hibbs, D. E. Crop yield and tree-leaf production in three planting patterns of temperate-zone alley cropping in Oregon, USA. *Agroforest Syst* **46**, 273–288 (1999).

14 Eichhorn, M. P. *et al.* Silvoarable systems in europe - past, present and future prospects. *Agroforest Syst* **67**, 29–50 (2006).

15 Beyer, R. M., Hua, F. Y., Martin, P. A., Manica, A. & Rademacher, T. Relocating croplands could drastically reduce the environmental impacts of global food production. *Communications Earth & Environment* **3** (2022).

16 Zheng, Q. M. *et al.* The neglected role of abandoned cropland in supporting both food security and climate change mitigation. *Nature Communications* **14** (2023).

17 Næss, J. S., Cavalett, O. & Cherubini, F. The land-energy-water nexus of global bioenergy potentials from abandoned cropland. *Nature Sustainability* **4**, 525–+ (2021).

18 Grogan, D., Frolking, S., Wisser, D., Prusevich, A. & Glidden, S. Global gridded crop harvested area, production, yield, and monthly physical area data circa 2015. *Scientific Data* **9** (2022).

19 Mauser, W. *et al.* Global biomass production potentials exceed expected future demand without the need for cropland expansion. *Nature Communications* **6** (2015).

20 Zhong, H. L. *et al.* Global spillover effects of the European Green Deal and plausible mitigation options. *Nature Sustainability* (2024).

21 Cook-Patton, S. C. *et al.* Mapping carbon accumulation potential from global natural forest regrowth. *Nature* **585**, 545–+ (2020).
22 Kindermann, G. E., Schörghuber, S., Linkosalo, T. et al. Potential stocks and increments of woody biomass in the European Union under different management and climate scenarios. *Carbon Balance Manage* **8** (2013).
23 Gusti, M. K., G. An approach to modeling landuse change and forest management on a global scale. *in Proceedings of 1st International Conference on Simulation and Modeling Methodologies, Technologies and Applications (SciTePress - Science and and Technology Publications, 2011)* (2011).
24 Forsell, N. *et al.* Impact of modelling choices on setting the reference levels for the EU forest carbon sinks: how do different assumptions affect the country-specific forest reference levels? *Carbon Bal Manage* **14** (2019).
25 Amadu, F. O., Miller, D. C. & McNamara, P. E. Agroforestry as a pathway to agricultural yield impacts in climate-smart agriculture investments: Evidence from southern Malawi. *Ecol Econ* **167** (2020).
26 Avitabile, V. & Camia, A. An assessment of forest biomass maps in Europe using harmonized national statistics and inventory plots. *For Ecol Manage* **409**, 489–498 (2018).
27 Boettcher, H., Verkerk, P. J., Gusti, M., Havlík, P. & Grassi, G. Projection of the future EU forest CO2 sink as affected by recent bioenergy policies using two advanced forest management models. *Gcb Bioenergy* **4**, 773–783 (2012).
28 De Rosa, D. *et al.* Soil organic carbon stocks in European croplands and grasslands: How much have we lost in the past decade? *Global Change Biol* **30** (2024).
29 Ledo, A. *et al.* Changes in soil organic carbon under perennial crops. *Global Change Biol* **26**, 4158–4168 (2020).
30 Bacar, T., Cheng, Y. , Wang, Y. , Kaboul, K. and Lopes, N. The Effect of Vegetation Restoration in Soil Organic Carbon Storage. *Open Journal of Soil Science* **12**, 427–445 (2022).
31 Shi, S. W., Zhang, W., Zhang, P., Yu, Y. Q. & Ding, F. A synthesis of change in deep soil organic carbon stores with afforestation of agricultural soils. *Forest Ecol Manag* **296**, 53–63 (2013).
32 Bárcena, T. G. *et al.* Soil carbon stock change following afforestation in Northern Europe: a meta-analysis. *Global Change Biol* **20**, 2393–2405 (2014).
33 Hong, S. B. *et al.* Divergent responses of soil organic carbon to afforestation. *Nature Sustainability* **3**, 694–+ (2020).
34 Gvein, M. H. *et al.* Potential of land-based climate change mitigation strategies on abandoned cropland. *Communications Earth & Environment* **4** (2023).
35 Crawford, C. L., Wiebe, R. A., Yin, H., Radeloff, V. C. & Wilcove, D. S. Biodiversity consequences of cropland abandonment. *Nature Sustainability* (2024).
36 Folberth, C. *et al.* The global cropland-sparing potential of high-yield farming. *Nature Sustainability* **3**, 281–289 (2020).
37 Phalan, B. T. What Have We Learned from the Land Sparing-sharing Model? *Sustainability-Basel* **10** (2018).
38 Yu, Q. Y. *et al.* A cultivated planet in 2010-Part 2: The global gridded agricultural-production maps. *Earth Syst Sci Data* **12**, 3545–3572 (2020).
39 European Space Agency (ESA). Land Cover Climate Change Initiative (CCI) Product User Guide Version 2. . (2017).
40 Kindermann, G. E., Mcallum, I., Fritz, S. & Obersteiner, M. A global forest growing stock, biomass and carbon map based on FAO statistics. *Silva Fenn* **42**, 387–396 (2008).

41 Torralba, M., Fagerholm, N., Burgess, P. J., Moreno, G. & Plieninger, T. Do European agroforestry systems enhance biodiversity and ecosystem services? A meta-analysis. *Agr Ecosyst Environ* **230**, 150–161 (2016).
42 Graves, A. R. *et al.* Development and application of bio-economic modelling to compare silvoarable, arable, and forestry systems in three European countries. *Ecol Eng* **29**, 434–449 (2007).
43 Sereke, F., Graves, A. R., Dux, D., Palma, J. H. N. & Herzog, F. Innovative agroecosystem goods and services: key profitability drivers in Swiss agroforestry. *Agron Sustain Dev* **35**, 759–770 (2015).
44 Lovell, S. T. *et al.* Temperate agroforestry research: considering multifunctional woody polycultures and the design of long-term field trials. *Agroforest Syst* **92**, 1397–1415 (2018).
45 Xu, Y., Lehmann, L. M., de Jalón, S. & Ghaley, B. B. Assessment of Productivity and Economic Viability of Combined Food and Energy (CFE) Production System in Denmark. *Energies* **12** (2019).
46 Kanzler, M., Böhm, C., Mirck, J., Schmitt, D. & Veste, M. Microclimate effects on evaporation and winter wheat (L.) yield within a temperate agroforestry system. *Agroforest Syst* **93**, 1821–1841 (2019).
47 Baier, C., Gross, A., Thevs, N. & Glaser, B. Effects of agroforestry on grain yield of maize (L.)-A global meta-analysis. *Front Sustain Food S* **7** (2023).
48 Roupsard, O. *et al.* How far does the tree affect the crop in agroforestry? New spatial analysis methods in a parkland. *Agr Ecosyst Environ* **296** (2020).
49 FAO. *FAOSTAT Statistical Database*. https://www.fao.org/statistics/en
50 Koch, W., Czop, M., Nawrocka, A. & Wiacek, D. Contribution of Major Groups of Food Products to the Daily Intake of Selected Elements-Results from Analytical Determinations Supported by Chemometric Analysis. *Nutrients* **12** (2020).
51 Department for Environment Food and Rural Affairs. Landscape Recovery Scheme. (UK Government, 2022).
52 Uk Parliament. Agriculture Act 2020 & Environmental Land Management (ELM) Schemes. (2020).
53 Bmel. Rahmenplan der Gemeinschaftsaufgabe "Verbesserung der Agrarstruktur und des Küstenschutzes" (GAK) 2024–2027. (2023).
54 Bundesministerium für Umwelt, N., nukleare Sicherheit und Verbraucherschutz. *Aktionsprogramm Natürlicher Klimaschutz*. (Bundesministerium für Umwelt, Naturschutz, nukleare Sicherheit und Verbraucherschutz, 2023).
55 Ministère de la Transition, é. Stratégie Nationale Bas-Carbone (SNBC2). (2020).
56 Ministère de la Transition, é. Méthode Haies – Label Bas-Carbone. (2021).
57 Lasy, P. Forests in Poland: National Programme for Increasing the Forest Cover (KPZL). (2018).
58 Ministero dell'Agricoltura, d. S. A. e. d. F. Strategia Forestale Nazionale. (2022).
59 Ministerio para la Transición Ecológica y el Reto, D. Plan Forestal Español 2022–2032. (2022).
60 Regeringen, L. F., Danmarks Naturfredningsforening, Fødevareforbundet NNF, Dansk Metal, Dansk Industri, Kommunernes Landsforening,. *Aftale om et Grønt Danmark*. (Government of Denmark, 2021).
61 Government of the Netherlands. “Food-Forest” Green Deal. (2017).
62 Government of Romania. PNRR State-Aid Scheme (OUG 35/2022). (2022).
63 Scottish Government. Forestry Grant Scheme – Agroforestry Option. (2024).
64 Department of Agriculture , F. a. M. Native Tree Area Scheme. (2023).
65 van Eupen, M., Elbersen, B., Hazeu, G., Verzandvoort, S., Meijninger, W., & Mantel, S. Mapping the extent of marginal lands in Europe. https://www.midas-bioeconomy.eu/news/mapping-the-extent-of-marginal-land-in-europe/

66 Eurostat. Agri-environmental indicator - soil erosion. (2020). https://ec.europa.eu/eurostat/statistics-explained/index.php?title=Agri-environmental_indicator_-_soil_erosion

67 Estel, S., Kuemmerle, T., Levers, C., Baumann, M. & Hostert, P. Mapping cropland-use intensity across Europe using MODIS NDVI time series. *Environ Res Lett* **11** (2016).

68 Pravalie, R. *et al.* A unifying modelling of multiple land degradation pathways in Europe. *Nature Communications* **15** (2024).

69 Griscom, B. W. *et al.* Natural climate solutions. *P Natl Acad Sci USA* **114**, 11645–11650 (2017).

70 Bernal, B., Murray, L. T. & Pearson, T. R. H. Global carbon dioxide removal rates from forest landscape restoration activities. *Carbon Bal Manage* **13** (2018).

71 Lindeskog, M. *et al.* Accounting for forest management in the estimation of forest carbon balance using the dynamic vegetation model LPJ-GUESS (v4.0, r9710): implementation and evaluation of simulations for Europe. *Geosci Model Dev* **14**, 6071–6112 (2021).

72 Schierhorn, F. *et al.* Post-Soviet cropland abandonment and carbon sequestration in European Russia, Ukraine, and Belarus. *Global Biogeochem Cy* **27**, 1175–1185 (2013).

73 Lu, N. *et al.* Biophysical and economic constraints on China's natural climate solutions. *Nature Climate Change* **12**, 847–+ (2022).

74 McMahon, S. M., Parker, G. G. & Miller, D. R. Evidence for a recent increase in forest growth. *P Natl Acad Sci USA* **107**, 3611–3615 (2010).

75 Pearson, J. A., Knight, D. H. & Fahey, T. J. Biomass and Nutrient Accumulation during Stand Development in Wyoming Lodgepole Pine Forests. *Ecology* **68**, 1966–1973 (1987).

76 Hasegawa, T., Fujimori, S., Ito, A. & Takahashi, K. Careful selection of forest types in afforestation can increase carbon sequestration by 25% without compromising sustainability. *Communications Earth & Environment* **5** (2024).

77 Krause, A., Knoke, T. & Rammig, A. A regional assessment of land-based carbon mitigation potentials: Bioenergy, BECCS, reforestation, and forest management. *Gcb Bioenergy* **12**, 346–360 (2020). 78 Tupek, B. *et al.* Extensification and afforestation of cultivated mineral soil for climate change mitigation in Finland. *Forest Ecol Manag* **501** (2021).

79 Burke, T., Rowland, C. S., Whyatt, J. D., Blackburn, G. A. & Abbatt, J. Spatially targeting national-scale afforestation for multiple ecosystem services. *Appl Geogr* **159** (2023).

80 Newbold, T. *et al.* Global effects of land use on local terrestrial biodiversity. *Nature* **520**, 45–+ (2015).

81 Hua, F. Y. *et al.* Opportunities for biodiversity gains under the world's largest reforestation programme. *Nature Communications* **7** (2016).

82 Jeanneret, P. *et al.* An increase in food production in Europe could dramatically affect farmland biodiversity. *Communications Earth & Environment* **2** (2021).

83 Ceaușu, S., Leclère, D. & Newbold, T. Geography and availability of natural habitat determine whether cropland intensification or expansion is more detrimental to biodiversity. *Nature Ecology & Evolution* (2025).

84 Ríos-Touma, B. *et al.* Biodiversity responses to land-use change in the equatorial Andes. *Ecol Indic* **156** (2023).

85 Kamp, J. *et al.* Farmland bird responses to land abandonment in Western Siberia. *Agr Ecosyst Environ* **268**, 61–69 (2018).

86 Newbold, T., Oppenheimer, P., Etard, A. & Williams, J. J. Tropical and Mediterranean biodiversity is disproportionately sensitive to land-use and climate change. *Nature Ecology & Evolution* **4**, 1630–1638 (2020).

87 Wang, W. D., Pijl, A. & Tarolli, P. Future climate-zone shifts are threatening steep-slope agriculture. *Nature Food* **3**, 193–196 (2022).
88 Jarasiunas, G. Assessment of the agricultural land under steep slope in Lithuania. *Journal of Central European Agriculture* **17**, 176–187 (2016).
89 Drees, L. R. *et al.* Steepland resources: characteristics, stability and micromorphology. *Catena* **54**, 619–636 (2003).
90 Shaxson, F. New concepts and approaches to land management in the tropics with emphasis on steeplands. *Food and Agriculture Organization of the United Nations (FAO)* (1999).
91 Posner, J. L. & Mcpherson, M. F. Agriculture on the Steep Slopes of Tropical America - Current Situation and Prospects for the Year 2000. *World Dev* **10**, 341–353 (1982).
