# Supplementary material for: Reconciling crop production, climate action and nature conservation in Europe by agricultural intensification and extensification
Source: Nat Commun. 2025 Nov 21;16:10289. doi: 10.1038/s41467-025-65201-4 (PMC12638777; doi:10.1038/s41467-025-65201-4)
Supplement: Supplementary file 2 — Reporting Summary [file 41467_2025_65201_MOESM2_ESM.pdf]

nature portfolio

Corresponding author(s): Ting Hua

Last updated by author(s): Sep 6, 2025

Reporting Summary

Nature Portfolio wishes to improve the reproducibility of the work that we publish. This form provides structure for consistency and transparency in reporting. For further information on Nature Portfolio policies, see our Editorial Policies and the Editorial Policy Checklist.

Statistics

For all statistical analyses, confirm that the following items are present in the figure legend, table legend, main text, or Methods section.

n/a	Confirmed	
☐	☒	The exact sample size (*n*) for each experimental group/condition, given as a discrete number and unit of measurement
☒	☐	A statement on whether measurements were taken from distinct samples or whether the same sample was measured repeatedly
☒	☐	The statistical test(s) used AND whether they are one- or two-sided<br>*Only common tests should be described solely by name; describe more complex techniques in the Methods section.*
☒	☐	A description of all covariates tested
☒	☐	A description of any assumptions or corrections, such as tests of normality and adjustment for multiple comparisons
☐	☒	A full description of the statistical parameters including central tendency (e.g. means) or other basic estimates (e.g. regression coefficient) AND variation (e.g. standard deviation) or associated estimates of uncertainty (e.g. confidence intervals)
☒	☐	For null hypothesis testing, the test statistic (e.g. *F*, *t*, *r*) with confidence intervals, effect sizes, degrees of freedom and *P* value noted<br>*Give P values as exact values whenever suitable.*
☒	☐	For Bayesian analysis, information on the choice of priors and Markov chain Monte Carlo settings
☒	☐	For hierarchical and complex designs, identification of the appropriate level for tests and full reporting of outcomes
☒	☐	Estimates of effect sizes (e.g. Cohen's *d*, Pearson's *r*), indicating how they were calculated

Our web collection on statistics for biologists contains articles on many of the points above.

Software and code

Policy information about availability of computer code

Data collection	All input data used in this study are publicly available from the corresponding references.
Data analysis	Data analysis in this study are conducted at ArcGIS and Matlab.

For manuscripts utilizing custom algorithms or software that are central to the research but not yet described in published literature, software must be made available to editors and reviewers. We strongly encourage code deposition in a community repository (e.g. GitHub). See the Nature Portfolio guidelines for submitting code & software for further information.

Data

Policy information about availability of data

All manuscripts must include a data availability statement. This statement should provide the following information, where applicable:

- Accession codes, unique identifiers, or web links for publicly available datasets
- A description of any restrictions on data availability
- For clinical datasets or third party data, please ensure that the statement adheres to our policy

Datasets used in this analysis are publicly available from the references provided within the paper. Source data is provided with this article and our modeling script and outputs are available at https://doi.org/10.5281/zenodo.16881974.

Research involving human participants, their data, or biological material

Policy information about studies with human participants or human data. See also policy information about sex, gender (identity/presentation), and sexual orientation and race, ethnicity and racism.

Reporting on sex and gender	n.a.
Reporting on race, ethnicity, or other socially relevant groupings	n.a.
Population characteristics	n.a.
Recruitment	n.a.
Ethics oversight	n.a.

Note that full information on the approval of the study protocol must also be provided in the manuscript.

Field-specific reporting

Please select the one below that is the best fit for your research. If you are not sure, read the appropriate sections before making your selection.

☐ Life sciences ☐ Behavioural & social sciences ☒ Ecological, evolutionary & environmental sciences

For a reference copy of the document with all sections, see nature.com/documents/nr-reporting-summary-flat.pdf

Ecological, evolutionary & environmental sciences study design

All studies must disclose on these points even when the disclosure is negative.

Study description	This study identifies suboptimal cropland for revegetation across Europe to support achievement of climate and nature conservation goals, while investigating various strategies to preserve total crop production and quantifying the overall benefits and trade-offs for climate change mitigation and biodiversity conservation. Eight scenarios are designed, combining cropland use modes (intensification or extensification), crop switching strategies (crops with the highest calorie supply or local suitability), and revegetation options of suboptimal cropland (natural vegetation regrowth or active afforestation).
Research sample	We use existing geo-spatial data products. All data are available publicly from the corresponding references
Sampling strategy	For information about data sampling, see previous relevant publications for specific datasets
Data collection	We use existing geo-spatial data products. All data are available publicly from the corresponding references
Timing and spatial scale	2022. Europe. ~300 m spatial resolution, 5 min spatial resolution
Data exclusions	No data were excluded from the analyses
Reproducibility	The study is fully reproducible by acquiring necessary datasets and applying the same methodology
Randomization	Randomization is not relevant for this study
Blinding	Blinding is not relevant for this study

Did the study involve field work? ☐ Yes ☒ No

Reporting for specific materials, systems and methods

We require information from authors about some types of materials, experimental systems and methods used in many studies. Here, indicate whether each material, system or method listed is relevant to your study. If you are not sure if a list item applies to your research, read the appropriate section before selecting a response.

Materials & experimental systems

n/a	Involved in the study
☒	☐ Antibodies
☒	☐ Eukaryotic cell lines
☒	☐ Palaeontology and archaeology
☒	☐ Animals and other organisms
☒	☐ Clinical data
☒	☐ Dual use research of concern
☒	☐ Plants

Methods

n/a	Involved in the study
☒	☐ ChIP-seq
☒	☐ Flow cytometry
☒	☐ MRI-based neuroimaging

Plants

Seed stocks	n.a.
Novel plant genotypes	n.a.
Authentication	n.a.
